# miR-29b-3p suppresses the malignant biological behaviors of AML cells via inhibiting NF-κB and JAK/STAT signaling pathways by targeting HuR

**DOI:** 10.1186/s12885-022-09996-1

**Published:** 2022-08-20

**Authors:** Yong-Jing Tang, Wei Wu, Qiao-Qian Chen, Shu-Han Liu, Zhi-Yuan Zheng, Zhao-Lei Cui, Jian-Ping Xu, Yan Xue, Dong-Hong Lin

**Affiliations:** 1 Department of Laboratory Medicine, The School of Medical Technology and Engineering, Fujian Medical University, Fuzhou, 350004 Fujian China; 2 Department of Dermatology, The Third People’s Hospital of Hangzhou, Hangzhou, 310009 Zhejiang China; 3 Medical Technology Experimental Teaching Center, The School of Medical Technology and Engineering, Fujian Medical University, Fuzhou, 350004 Fujian China; 4 Department of Clinical Laboratory, Fujian Provincial Cancer Hospital, Fuzhou, 350014 Fujian China

**Keywords:** HuR, miR-29b-3p, Malignant biological behaviors, NF-κB and JAK/STAT signaling pathways, Acute myeloid leukemia

## Abstract

**Background:**

HuR/ELAVL1 (embryonic lethal abnormal vision 1) was a downstream target of miR-29b in some cancer cells. HuR protein exerts important prognostic effects of involving in the pathogenesis and development of acute myeloid leukemia (AML). This study aims to investigate the role of miR-29b-3p in biological behaviors of AML cells by targeting HuR and the involvement of the NF-κB and JAK/STAT signaling pathways.

**Methods:**

The expressions of HuR and miR-29b-3p in AML cells were determined using RT-qPCR and Western blot, and the association between them was analyzed using the Spearman method. Next, the target relationship between HuR and miR-29b-3p was predicted by biological information databases and verified by the dual-luciferase reporter gene assay. MTS, methyl cellulose, flow cytometry and transwell assay were employed to detect the cell proliferation, clone formation, cell cycle and apoptosis, invasion and migration respectively, the effect of miR-29b-3p targeted HuR on the biological behaviors of AML cells was explored after over- /down-expression of miR-29b-3p and rescue experiment. Then, immunofluorescence assay and western blot were employed to detect location expression and phosphorylation levels of NF-κB and JAK/STAT signaling pathways related molecules respectively.

**Results:**

HuR was negatively correlated with miR-29b-3p, and was the downstream target of miR-29b-3p in AML cells. When miR-29b-3p was overexpressed in AML cells, HuR was down-regulated, accompanied by cell viability decreased, cell cycle arrest, apoptosis increased, invasion and migration weakened. Moreover, the opposite result appeared after miR-29b-3p was down-regulated. The rescue experiment showed that miR-29b-3p inhibitor could reverse the biological effect of HuR down-regulation in AML cells. Molecular pathway results showed that miR-29b-3p could inhibit p65 expression in nucleus and phosphorylation levels of p65, IκBα, STAT1, STAT3 and STAT5.

**Conclusion:**

miR-29b-3p can inhibit malignant biological behaviors of AML cells via the inactivation of the NF-κB and JAK/STAT signaling pathways by targeting HuR. miR-29b-3p and its target HuR can be used as a new potential molecular for AML treatment.

**Supplementary Information:**

The online version contains supplementary material available at 10.1186/s12885-022-09996-1.

## Background

HuR/ELAVL1 (also called ELAV-like protein 1) is encoded by ELAVL1 gene located on chromosome 19p13.2, and it ubiquitously expressed in mammals and functionally involved in modulating mRNA stability and translational efficiency [1, 2]. It stabilizes or destabilizes mRNAs which are closely related to tumor progression including the cell cycle disorder, excessive cell proliferation and invasion, and cell resistance to apoptosis [2]. Previous clinical studies have demonstrated that HuR is associated with lymph node metastasis in malignant tumors [3–5]. HuR over-expression has been detected in almost all types of cancer tissues, including acute myeloid leukemia (AML) as well [6]. One of the mechanisms to restrain AML is the inhibition of a key protein nuclear factor κB (NF-κB) with the involvement of the JAK/STAT signaling pathway, which transcriptionally regulates stress-response genes. IκBα mRNA has a long AU-rich 3′UTR (Untranslated Regions) containing a number of predicted hits that target a previously identified HuR motif. A previous study reported that IκBα 3′UTR transcripts were specifically associated with HuR, and HuR over-expression increased IκB-α protein levels, which in turn downregulated NF-κB in the nucleus [7]. Moreover, HuR has been reported to destabilize STAT3 and STAT5 mRNAs [8], which implies the underlying involvement of the JAK/STAT signaling pathway. Thus, elucidating the regulation of HuR expression is critical for a better understanding of the molecular mechanism behind the pathogenesis of AML.

Recently, post-transcriptional regulators of gene expression have gained solicitous attentions. MicroRNAs (miRNAs) are a class of endogenous highly conserved non-coding small molecule RNAs with approximately 19-25 nucleotides. Previous studies proved that miRNAs involved in the regulation of genes related to the hematopoietic system via a complex regulatory network [9–11]. Strikingly, abnormal miRNA expressions have been observed in cases of AML as well. For instance, miR-29b over-expression has significantly suppressed the development of AML [12]. In addition, it was found that miR-29b as a tumor suppressor is down-expressed in AML cell lines and primary AML blasts, and induces cell apoptosis in tumor cells and dramatically reduces tumorigenicity in a xenograft leukemia model [13]. Previous studies reported that HuR was a downstream target of miR-29b in some cancer cells: miR-29b inhibits expression of HuR post-transcriptionally, thus playing a role in the regulation of intestinal epithelial cells (IECs) proliferation and intestinal epithelial homoeostasis [14]; miR-29 interacted directly with HuR, decreased miR-29 abundance correlated with increased activity of NF-κB in sarcoma cell lines [15]. Currently, whether HuR is regulated by miR-29b in AML and the mechanism of their interactions are still unclear.

In this study, we predicted that HuR 3′UTR had some conservative targets of miR-29b using bioinformatics resources. We aimed to explore the effects of miR-29b-3p on malignant biological behaviors of AML and the involvement of the NF-κB and JAK/STAT signaling pathways by targeting HuR, in the hope of providing a novel molecular target for the treatment of AML. We demonstrate for the first time that HuR is the direct target of miR-29b-3p, responsible for excessive cell proliferation and resistance to apoptosis by mediating activities of the NF-κB and JAK/STAT pathways in the pathogenesis of AML.

## Methods

### Cell lines and cultures

Human myeloid leukemia cell lines including K562, NB4, U937, HL-60 and imatinib-resistant K562/G01 and HEK 293T cells were selected from CCTCC (China Center for Type Culture Collection, Wuhan, China) and cultured in RPMI-1640 medium supplemented with 10% fetal bovine serum (FBS). Myeloid leukemia cell line Kasumi-1 (kindly provided by Prof. Ligen Liu, the Fifth People's Hospital of Shanghai, Fudan University, Shanghai, China) was cultured in RPMI-1640 medium supplemented with 15% FBS. K562/G01 cells grown in an imatinib-free culture medium (4 μM) for at least two weeks for each experiment. HEK 293T cell line was cultured in DMEM (Invitrogen) containing 1% penicillin/streptomycin and 10% FBS. All cell lines were incubated at 5% CO_2_ and 37℃.

### Construction of miR-29b-3p over- and down-expression vectors

The lentivirus vector containing overexpressed and scrambled miR-29b-3p was constructed by GeneChem Company (Shanghai, China), the sequences of miR-29b-3p synthetic vectors as following: 5'-UAGCACCAUUUGAAAUCAGUGUU-3'. miR-29b-3p mimic, inhibitor and relevant negative controls were synthesized by GenePharma Company (Shanghai, China), the sequences of synthetic vectors as following: miR-29b-3p mimic: 5'-UAGCACCAUUUGAAAUCAGUGUU-3', miR-29b-3p inhibitor: 5'-AACACUGAUUUCAAAUGGUGCUA-3'.

### Cell transfection

According to the lentivirus infection protocol, the optimal infection conditions of K562 and U937 cells respectively included the multiplicity of infection were 20 and 50, supplemented with enhancer infection supplement (ENI.S.) and polybrene. Stably infected cell lines were selected with 1.7 μg/ml puromycin (Sigma-Aldrich). Then the cells were divided into the miR-29b-3p group (transfected with a miR-29b-3p lentivirus vector), the NC group (transfected with a scramble-miR-29b-3p lentivirus vector) and the CON group (blank control). miR-29b-3p stably transfected cells were harvested at 96h post-infection for various assays subsequently reported in this study.

Electric transfection reagents containing cells transfected with miR-29b-3p mimics, inhibitor or negative controls using the TransEasy electrical transfection kit (Cellapy Biotechnology, China) according to the manufacturer’s instruction were added to electrode cups, which were placed in an X Unit (Lonza Nucleofector™4D, Switzerland) to switch on the procedure. The transfected cells were incubated at 5% CO_2_ and 37℃ for 48h.

### Dual-luciferase reporter gene assay

HuR-3′UTR luciferase reporter plasmids with wild-type HuR-3′UTR (wt UTR) and mutated HuR-3′UTR (mut UTR) in the predicted miR-29b-3p binding site were constructed by GenePharma (Shanghai, China), the sequences of synthetic vectors as following: HuR wt: 5'-CTCTAGTCGCAGCTCTGTGACTGATTCCCTCCCGGGTGCTGAGTCCCCTCCCCGGCCACC-3', 

HuR mut: 5'-CTCTAGTCGCAGCTCTGTGACTGATTCCCTCCCGCCACGAGAGTCCCCTCCCCGGCCACC-3'. The dual luciferase reporter plasmids with HuR wt UTR, or with mut UTR and miR-29b-3p or scramble mimics, which were co-transfected into HEK 293T cells using Lipofectamine 2000 (Invitrogen, USA) according to the manufacture’s instruction. After 48h of transfection, the firefly and renilla luciferase activities were measured using the Dual-Luciferase reporter assay system (Promega, USA). The relative luciferase unit (RLU) activity was determined as follows, with the cell lysate of the reporter gene regarded as the blank control and the Renilla luciferase as the internal control: RLU = RLU firefly luciferase / RLU Renilla luciferase.

### Real-time quantitative PCR analysis

Total RNAs were extracted using Trizol reagent according to the manufacturer’s protocol. The reverse transcriptase (RT) reactions of miR-29b-3p and HuR were respectively amplified using a Bluge-Loop^TM^ miRNA qRT Starter Kit (RiboBio, Guangzhou) and a RevertAid First Strand cDNA Synthesis Kit (Thermo Scientific^TM^). Quantitative PCR (qPCR) analyses for miR-29b-3p and HuR were performed on a 7500 Real-time PCR system (Thermo Fisher Scientific, Waltham, MA, USA), using a Bluge-Loop^TM^ miRNA qPCR Starter Kit (RiboBio, Guangzhou) and UltraSYBR Mixture (Low Rox), respectively. The primer sequences of HuR for qPCR were shown as flowing: Forward: 5'-GGCGCAGAGATTCAGGTTCT-3', Reverse: 5'-TCCTGCCCCAGGTTGTAGAT-3'. The relative quantification of miR-29b-3p and HuR expressions normalized to U6 or GAPDH was calculated using the comparative 2^-ΔΔCt^ method.

### Western blot analysis

Total protein was extracted from cells and lysed using RIPA buffer (Boster, USA), and the protein concentration was determined by the BCA Kit (Boster, USA). Nuclear and cytoplasmic extracts were prepared using the nuclear and cytoplasmic protein extraction kit (Cwbio, China) following the manufacturer’s protocol. Equal amounts from the cell lysates were separated by SDS-PAGE and transferred electrophoretically onto PVDF membranes. The membranes were cut prior to hybridisation with antibodies during blotting. After blocking, the membranes were incubated with the following specific primary antibodies overnight: anti-HuR (ab200342, Abcam), anti-p65 (ab32536,Abcam), anti-phospho-p65 (Ser536) (#3031S,CST), anti-IκBα(#9242S, CST), anti-phospho-IκBα (Ser32/36) (5A5) (#9246S, CST), anti-STAT1 (ab109320, Abcam), anti-phospho-STAT1(Y701) (ab30645,Abcam), anti-STAT3 (ab68153, Abcam), anti-phospho-STAT3 (Tyr705) (#8204, CST), anti-STAT5 (D3N2B) (#25656, CST), anti-phospho-STAT5 (Tyr694) (D47E4) (#4322, CST). Afterward, the membranes were incubated with the HRP-conjugated secondary antibody (SSA016, Sino biological) for 2h at room temperature. Solution A and solution B of ECL chemiluminescence kit were mixed in a ratio of 1:1 for one minute. The PVDF membrane was moved to the exposure table, and the chromogenic solution was dripping to cover the surface of the membrane, then put it into the Bio-Rad ChemiDoc XRS+ Chemiluminescence Imager for exposure. Protein bands were visualized and quantitated using the Image J 1.43 software (NIH, MD, USA), and data were normalized to GAPDH (#5174, CST) and PCNA (10205-2-AP, Proteintech).

### MTS assay

Cells were seeded to 96-well plates and were detected at different times points (24h, 48h, 72h and 96h) using the MTS assay (Promega, USA). The absorbance was measured at 492/630 nm using a Microplate Reader (MK3, Thermo Fisher Scientific, USA).

### Cell clone formation assay

The cells were cultured in 24-well plates in RPMI-1640 medium containing 1.6% methyl cellulose (Sigma, USA) for 7-10 days. The number of colonies (containing ≥ 40 cells) was counted and the efficiency of colony formation was assessed.

### Flow cytometry

Cell apoptosis was examined using Annexin V-PE and 7-AAD staining assays (BD Bioscience, USA) following the manufacturer’s protocols, and cell cycle was analyzed using a Cell Cycle Analysis Kit (Keygen Biotech, China). Cell cycle and apoptosis were detected by flow cytometry (FCM) analysis (Accuri C6, BD Bioscience, USA).

### Transwell assay

In-vitro invasion and migration were analyzed using 8 µm pore transwell chambers (Coring, MA, USA). The cells were reseeded onto the matrigel-coated upper chambers (Corning, USA) containing serum-free RPMI-1640, and 10% FBS was added to the lower chambers. Cells were incubated for 24h for invasion assay. For invasion assay, cells attaching to the lower surface of the membrane were fixed by methanol and stained with Wright-Giemsa, and the number of cells was counted under a light microscope (IX71, Olympus, Japan). For migration assay, cells migrating to the lower chambers were collected and detected using MTS.

### Immunofluorescence assay

Cells were fixed with 4% paraformaldehyde (DingGuo, Beijing, China) for 15min at room temperature, and permeabilized with 0.1%Triton X-100 (Sigma, USA) for 15 min. After blocking with 5% goat serum (Gibco, USA) for 2h at room temperature, cells were incubated with anti-p65 antibody (at a dilution of 1:200, Affinity Biosciences, USA) overnight at 4℃. Then cells were incubated with a 1:500 dilution of Alexa Fluor 555 fluorescein-labelled goat anti-rabbit IgG antibody (Dingjie, China) in darkness for 2h. After the cells for washing three times and stained with DAPI (Servicebio, Wuhan, Chian) for 5 min, images were captured by a camera on an inverted fluorescence microscope (Olympus, Japan).

### HuR knockdown and rescue experiment

To silence HuR expression, a small interfering RNA (siRNA) targeting HuR (5'-AAGAGGCAAUUACCAGUUUCA-3') and a control siRNA (5'-UUGUUCGAA CGUGUCACGUTT-3') were purchased from GenePharma Company (Shanghai, China). Three groups were established, which included HuR-KD group (transfected with HuR siRNA), HuR-NC group (transfected with control siRNA) and rescue group (co-transfected with HuR siRNA and miR-29b-3p inhibitor). All transfections were performed using Lipofectamine 2000 (Invitrogen, USA) following the manufacturer’s instructions. Then biological behaviors of AML cells including cell proliferation, clone formation, cell cycle and apoptosis, invasion and migration were carried out according to the above steps.

### Statistical analysis

All experiments were repeated at least three times. Data were expressed as mean± standard deviation (SD) using SPSS 24.0 statistical software. For variables with a normal distribution, comparisons between two groups and homogeneity of variance was verified using an independent samples t-test. Differences between multiple groups were compared using one-way ANOVA. For variables with a non-normal distribution, a nonparametric test was employed. Spearman’s coefficient was used for determining the correlation between two variables. A *P* value of <0.05 was considered statistically significant.

## Results

### HuR is overexpressed in AML cells and negatively correlated with miR-29b-3p

**Fig. 1:**
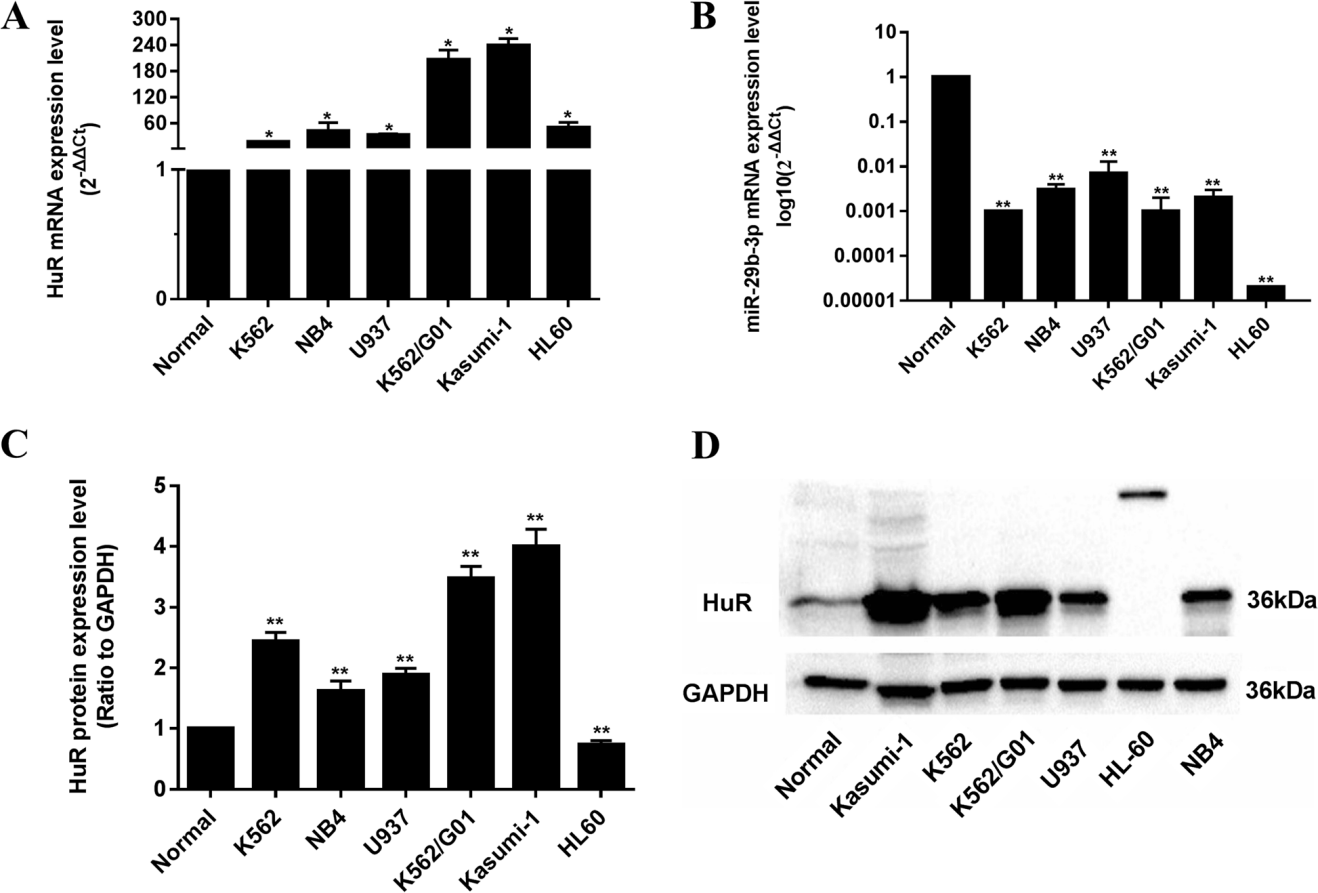
Expression of HuR and miR-29b-3p in AML cell lines detected by RT-qPCR and Western blot. **a**, **b** Expression of HuR and miR-29b-3p in mRNA level in AML cell lines, respectively. **c**, **d** Protein expression of HuR in AML cell lines, quantification of HuR was normalized to GAPDH. (*P<0.05, **P<0.01, vs. healthy normal control). The original blots/gels are presented in Supplementary Figure 1. Note: Normal, healthy normal control from normal human peripheral blood mononuclear cells. The experiments were repeated at least three times

The expressions of HuR and miR-29b-3p in AML cells were determined by RT-qPCR and Western blot analyses, and their relationship was analyzed. The results showed that HuR was overexpressed in 6 myeloid leukemia cell lines including K562, NB4, U937, Kasumi-1, HL-60 and imatinib resistant K562/G01 cells compared to the normal control (Fig. 1a), accompanied by miR-29b-3p downexpression (Fig. 1b). The correlation analysis showed that HuR mRNA expression was negatively correlated with miR-29b-3p expression in these cell lines (r=-0.829, *P*<0.05), so was HuR protein expression with miR-29b-3p expression (Fig. 1c-d). Owing to the miR-29b-3p is relatively moderately expressed in K562 and U937 cells, they were used in the following experiments.

### HuR is the directly target of miR-29b-3p

**Fig. 2:**
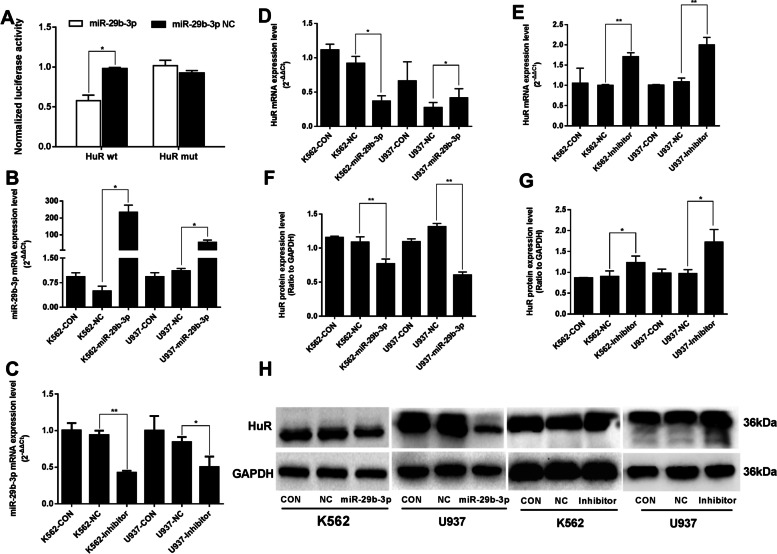
HuR was verified to be the downstream target of miR-29b-3p. **a** The dual luciferase activites assay of miR-29b-3p and HuR. **b**, **c **The mRNA expression levels of miR-29b-3p after up-regulating or inhibiting miR-29b-3p. **d**, **e** The mRNA expression of HuR after up-regulating or inhibiting miR-29b-3p. **f**-**h** Western blot of HuR expression were detected, quantification of HuR was normalized to GAPDH. (*P<0.05, **P<0.01, vs. NC group). The original blots/gels are presented in Supplementary Figure 2. The experiments were repeated at least three times

Putative binding sites in the HuR 3′UTR interacting with miR-29b-3p were predicted using microRNA.org, miRanda and RNAhybrid2.2 (data not shown). As shown in Fig. 2a, the luciferase activity of HuR wt UTR significantly decreased in miR-29b-3p transfected 293T cells (*P*<0.01). However, luciferase reporter activity was not significantly affected by HuR mut UTR (*P*>0.05). After miR-29b-3p over-expression (Fig. 2b) or inhibition (Fig. 2c), we validate the expression of HuR changes with miR-29b-3p expression. The results showed that HuR mRNA and protein levels were significantly lowered in the miR-29b-3p group and were elevated in the inhibitor group (Fig. 2d-h). The above results indicated that HuR was a downstream target of miR-29b-3p in AML cells.

### miR-29b-3p over-expression inhibits malignant biological behaviors of AML cells

**Fig. 3:**
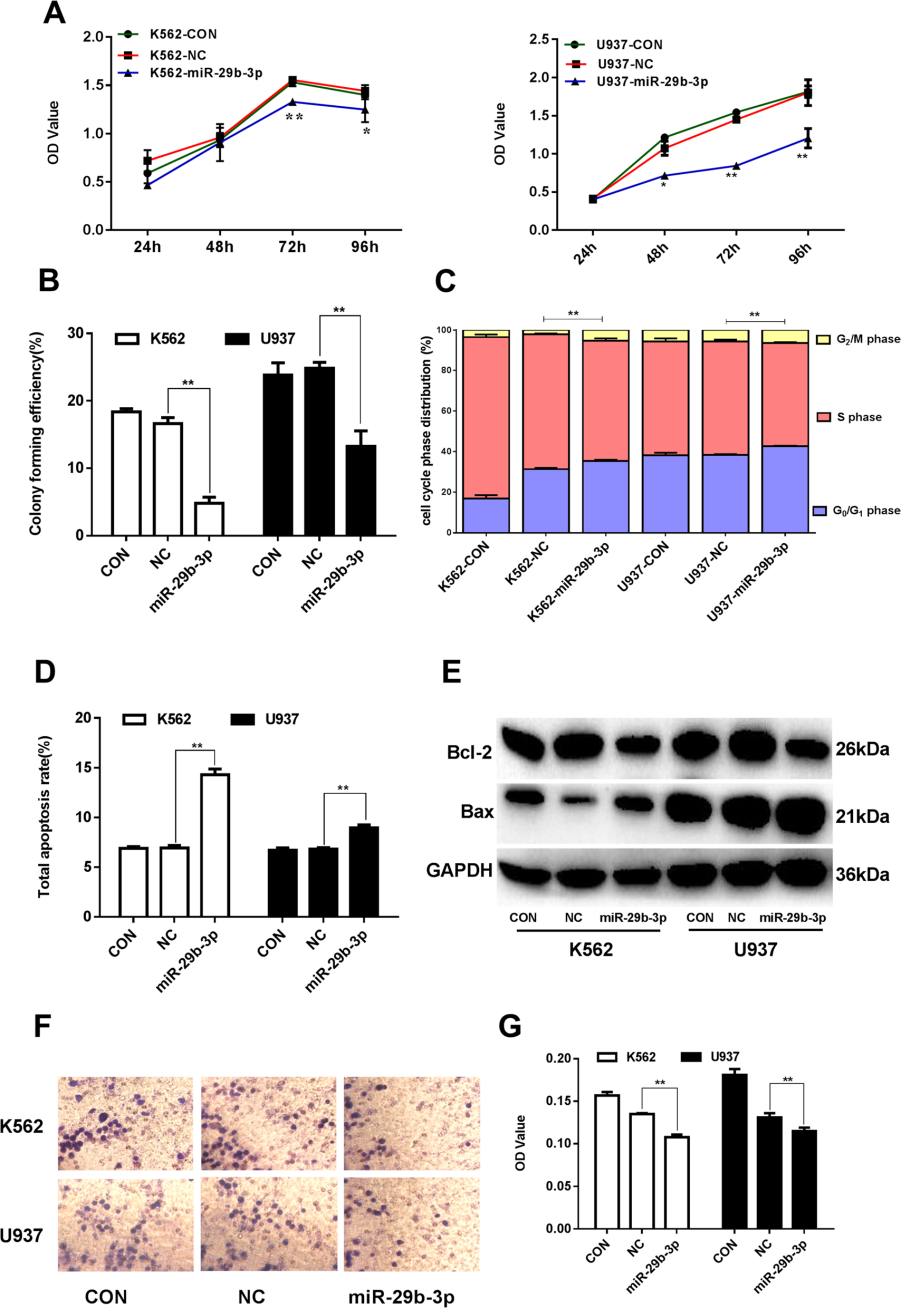
Biologic effects of over-expression miR-29b-3p evaluated in AML cells. **a** Grow curve of proliferation based on the OD value after miR-29b-3p restoration at different time points, resceptivly. **b** Colonies containing ≥40 cells were counted on day 7~10 using a microscope (×200). **c** Cells were labeled by PI and analyzed using FCM. The percentage of cells in G1/G0, S and G2/M of cell cycle were calculated. **d** Apoptotic cells were measured by FCM. The total apoptosis rate is equal to the sum of the early apoptosis rate and the late apoptosis rate. **e** The protein expression of Bcl-2 and Bax were detected by Western blot. The original blots/gels are presented in Supplementary Figure 3. Quantification of Bcl-2 and Bax were normalized to GAPDH and presented in Supplementary Figure 8a-c. **f** Wright-Giemsa stained invading cells were observed under microscope (×200). The invasion cell numbers were counted under microscope in five HP fields, its statistics are presented in Supplementary Figure 8j. **g** The OD values (proportional to cell numbers) of migrating cells were measured by MTS assay. (*P<0.05, **P<0.01, vs. NC group). The experiments were repeated at least three times

A restoration of miR-29b-3p expression resulted in a time-dependent inhibition of cell proliferation in K562 and U937 cells (Fig. 3a). The capability of colony formation of K562 and U937 cells significantly decreased (Fig. 3b). FCM analysis showed that the percentage of cells at the G0/G1 phase significantly increased in both the K562-miR-29b-3p group and U937-miR-29b-3p group (Fig. 3c, Supplementary Table 1). Additionally, the percentage of cells at the S phase significantly decreased in the K562-miR-29b-3p and U937-miR-29b-3p groups (*P*<0.01). These results suggested that miR-29b-3p over-expression in AML cells resulted in cell cycle arrest at the G0/G1 phase. As shown in Fig. 3d and Supplementary Table 4, the total apoptosis ratio was 14.300±0.000 in the K562-miR-29b-3p group and 8.896±0.289 in the U937-miR-29b-3p group, compared with the NC group (*P*<0.01), and the percentages of early apoptotic cells were 14.033±0.578% and 5.967±0.208%, respectively. Moreover, when miR-29b-3p over-expression, the anti-apoptotic protein Bcl-2 protein levels was lowered and the pro-apoptotic proteins Bax protein levels were elevated in K562 and U937 cells (*P*<0.01) (Fig. 3e, Supplementary Figure 8a-c). This indicated that cell apoptosis in AML cells was triggered by miR-29b-3p over-expression. The invading cells of miR-29b-3p groups were fewer than those in the paired NC groups in both K562 and U937 cells (*P*<0.01) (Fig. 3f, Supplementary Figure 8j). Furthermore, the OD value of migrating cells decreased in the K562-miR-29b-3 and U937-miR-29b-3p groups compared with the paired NC groups (*P*<0.01) (Fig. 3g). These results illustrate that the malignant biological behaviors of AML cells were inhibited after miR-29b-3p over-expression.

### Inhibition of miR-29b-3p promotes malignant biological behaviors of AML cells

**Fig. 4:**
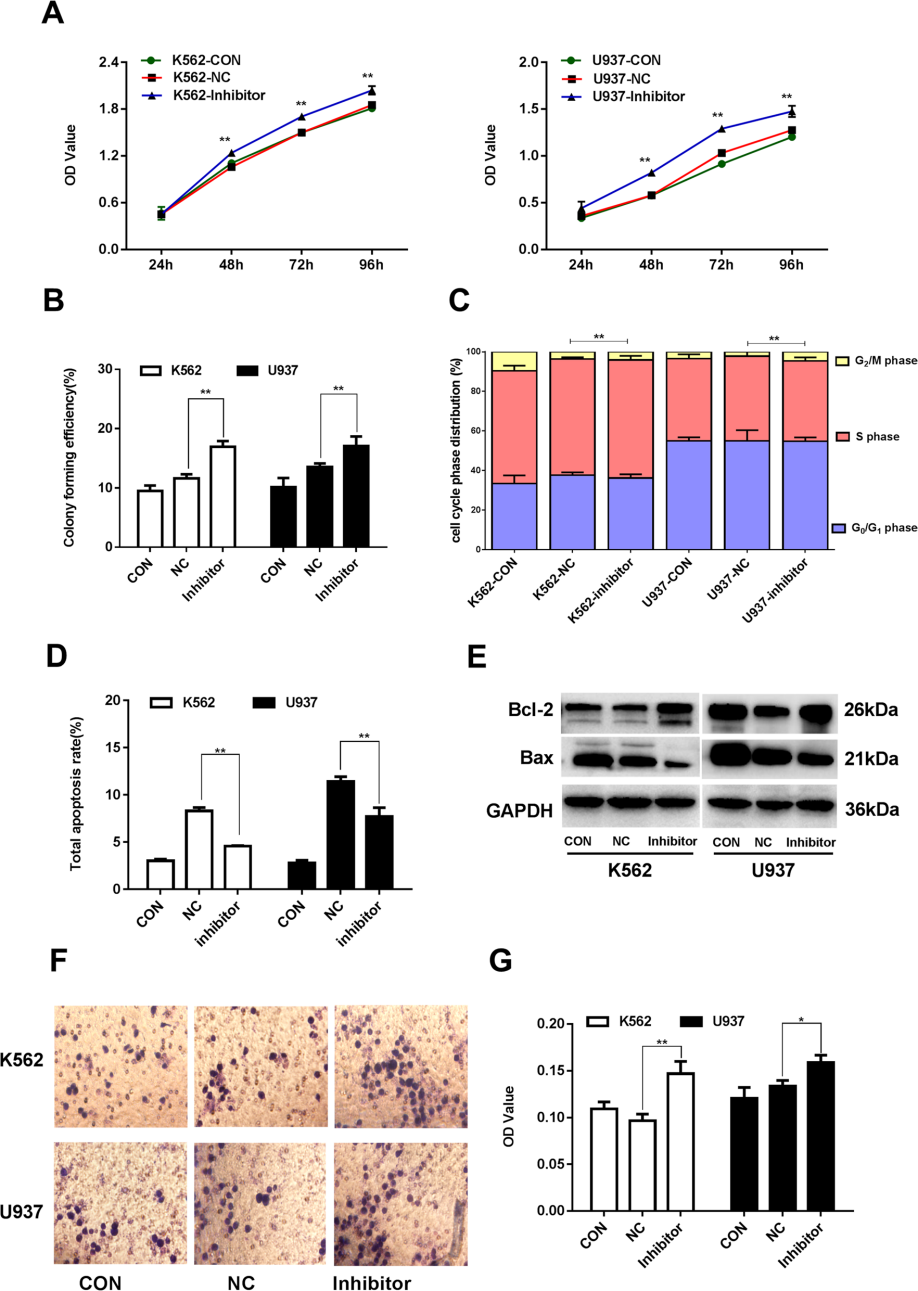
Biologic effects of inhibition miR-29b-3p evaluated in K562 and U937 cells. **a** Grow curve of proliferation based on the OD value after miR-29b-3p suppression at different time points, resepctively. **b** Colonies containing ≥40 cells were counted on day 7~10 using a microscope (×200). **c** Cells were labeled by PI and analyzed using FCM. The percentage of cells in G1/G0, S and G2/M of cell cycle were calculated. **d** Apoptotic cells were measured by FCM. The total apoptosis rate is equal to the sum of the early apoptosis rate and the late apoptosis rate. **e** The protein expression of Bcl-2 and Bax were detected by Western blot. The original blots/gels are presented in Supplementary Figure 4, quantification of Bcl-2 and Bax were normalized to GAPDH and presented in Supplementary Figure 8d-f. **f** Wright-Giemsa stained invading cells were observed under microscope (×200). The invasion cell numbers were counted under microscope in five HP fields, its statistics are presented in Supplementary Figure 8k. **g** The OD values (proportional to cell numbers) of migrating cells were measured by MTS assay. (*P<0.05, **P<0.01, vs. NC group). The experiments were repeated at least three times

When miR-29b-3p expression was suppressed by the inhibitor, cell proliferation and colony formation in both K562 and U937 cells were correspondingly promoted (Fig. 4a, b). As shown in Fig. 4c-e and Supplementary Table 2, 5 that miR-29b-3p inhibition didn’t affect the cell cycle, but promoted resistance to apoptosis in both K562 and U937 cells. Moreover, the invaded and migrated cells markedly increased in response to miR-29b-3p inhibition. The above data suggested that miR-29b-3p inhibition was able to promote cell proliferation, colony formation, as well as the invasion and migration abilities in AML cells (Fig. 4, Supplementary Figure 8d-f, 5k).

### HuR down-regulation inhibits malignant biological behaviors but reversed by miR-29b-3p inhibiton in AML cells

**Fig. 5:**
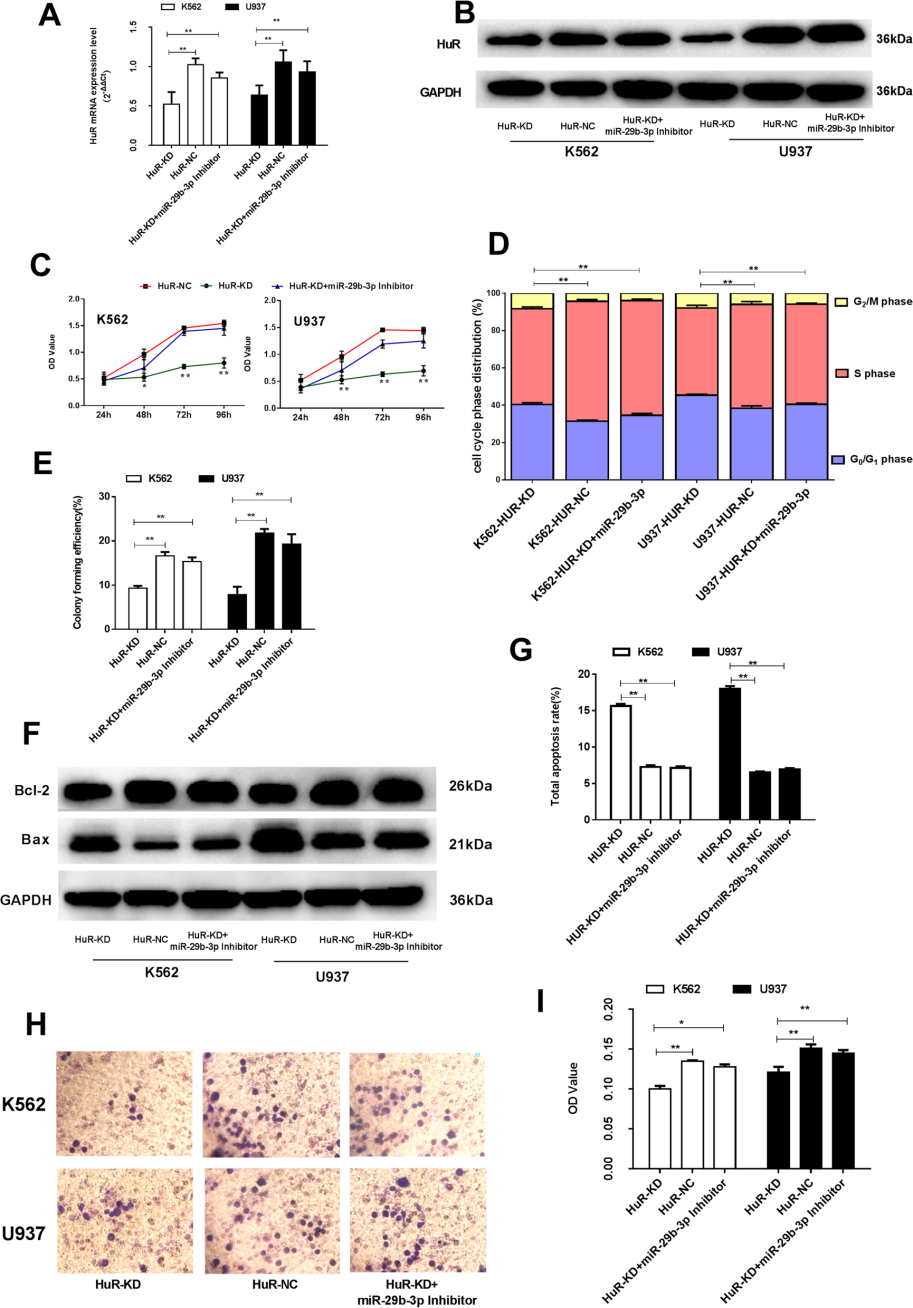
Biologic effects of HuR down-regulation and rescued by miR-29b-3p inhibitor evaluated in AML cells. **a** The mRNA expression of HuR after transfection with HuR siRNA and co-transfection with miR-29b-3p inhibitor. **b** Western blot of HuR expression were detected. The original blots/gels are presented in Supplementary Figure 5. **c** Grow curve of proliferation based on the OD value at different time points, resceptivly. **d** Cells were labeled by PI and analyzed using FCM. The percentage of cells in G1/G0, S and G2/M of cell cycle were calculated. **e** Colonies containing ≥40 cells were counted on day 7~10 using a microscope (×200). **f** The protein expression of Bcl-2 and Bax were detected by Western blot. The original blots/gels are presented in Supplementary Figure 5. Quantification of Bcl-2 and Bax were normalized to GAPDH and presented in Supplementary Figure 8g-i. **g** Apoptotic cells were measured by FCM. The total apoptosis rate is equal to the sum of the early apoptosis rate and the late apoptosis rate. **h** Wright-Giemsa stained invading cells were observed under microscope (×200). The invasion cell numbers were counted under microscope in five HP fields, its statistics are presented in Supplementary Figure 8l. **i** The OD values (proportional to cell numbers) of migrating cells were measured by MTS assay. (*P<0.05, **P<0.01). The experiments were repeated at least three times

In order to confirm whether the malignant biological behaviors inhibitory effect of miR-29b-3p in AML was mediated by HuR, we knocked down the HuR expression in K562 and U937 cells. RT-qPCR and Western blot analysis identified that after transfection with siRNA (Fig. 5a, b), HuR was down-regulated both in K562 and U937 cells (*P*<0.01). After transfection, cell proliferation and colony formation in both K562 and U937 cells were correspondingly decreased (*P*<0.01, Fig. 5c, e). Flow cytometry assay manifested that HuR down-regulation remarkably inhibited cell cycle at the G0/G1 phase of K562 and U937 cells, and promoted their apoptosis (*P*<0.01) (Fig. 5d, g, Supplementary Table 3,6). The Bcl-2 protein expression decreased and the Bax proteins expression levels increased in the cells due to knocking down the HuR expression (*P*<0.01) (Fig. 5f, Supplementary Figure 8g-i). In addition, the cell invasion and migration capabilities were markedly decreased in HuR knock down group (*P*<0.01) ( Fig. 5h, i, Supplementary Figure 8l). Through co-transfection of HuR siRNA and miR-29b-3p inhibitor to K562 and U937 cells for the rescue experiment. After co-transfection, HuR expression was correspondingly up-regulated both in K562 and U937 cells compared with HuR siRNA (Fig. 5a, b, *P*<0.01), we observed the opposite result of AML cell malignant biological behaviors from HuR down-regulation(Fig. 5c-i, *P*<0.01), the results signified that miR-29b-3p inhibitor could reverse the effect of HuR down-regulation in AML cells. These results further indicated that HuR was the direct functional target of miR-29b-3p in AML cells.

### miR-29b-3p inhibits the expression of p65 in nuclear and NF-κB signaling pathway in AML cells by targeting HuR

**Fig. 6:**
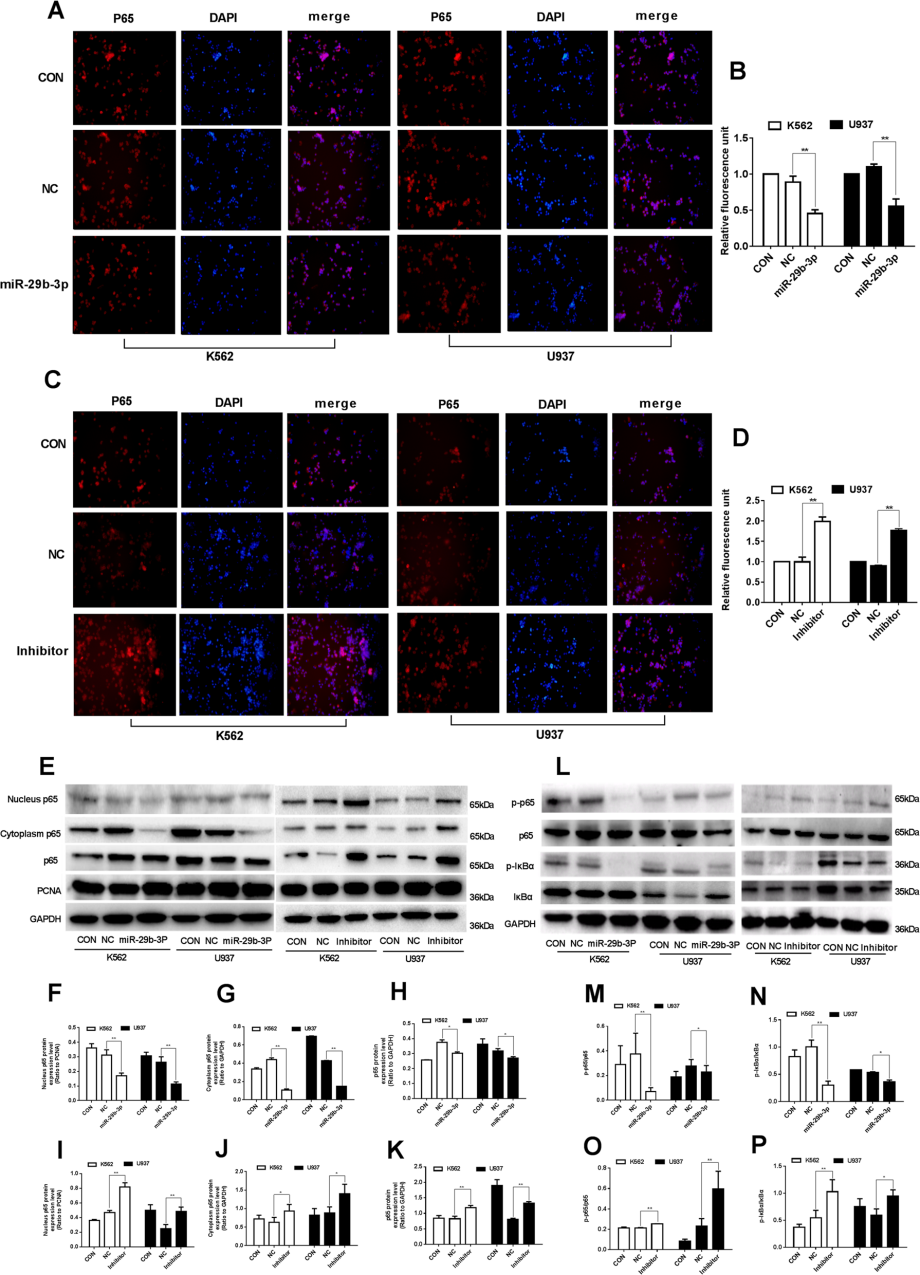
Effects of miR-29b-3p on the NF-κB signaling pathway protein by targeting HuR in AML cells. **a**, **c** Immunofluorescence experiment, fluorescent expressions of p65 were observed using fluorescence microscope (×200), images were longitudinally aligned represented as total pattern, nuclear and merge pattern, respectively. **b**, **d** Fluorescent expressions of merge pattern dividing by total pattern represented as the relative fluorescence rate of p65. **e** p65 at nuclear, cytosol and total cellular proteins were detected by western blot. **f**-**k** Quantification of nucleus p65 was normalized to PCNA, quantification of cytoplasm and total p65 were normalized to GAPDH. **l** p65, IκBα and their phosphorylation proteins expression were detected by western blot. The original blots/gels are presented in Supplementary Figure 6. **m**-**p** Quantifications of phosphorylated p65 and phosphorylated IκBα were normalized to p65 and IκBα after miR-29b-3p over-expression and inhibition, respectively. (*P<0.05, **P<0.01, vs. NC group). The experiments were repeated at least three times

According to the result of cellular immunofluorescence experiment as shown in Fig. 6, the relative fluorescence rate of p65 in K562 (0.230±0.007) and U937 (0.241±0.013) cells were significantly reduced in miR-29b-3p group compared with NC group (*P*<0.01) (Fig. 6a, b), while the fluorescence rate in K562 (1.986±0.113) and U937 (1.766±0.045) was increased after miR-29b-3p inhibited (*P*<0.01) (Fig. 6c, d). The result of Western blot confirmed the protein expression of p65 significant decrease in nuclear and cytoplasm in miR-29b-3p group in K562 and U937 cells (*P*<0.01), as shown in Fig. 6e-h. In order to further investigate the activity of p65, its phosphorylation protein expression was examined by Western blot (Fig. 6l, m). Consistently, miR-29b-3p suppressing the phosphorylation of p65 significantly (*P*<0.01) . In this case, the phosphorylation of IκBα was detected to further confirm the activation of NF-κB signaling pathway. The results indicated that the phosphorylation of IκBα was reduced (*P*<0.05) in miR-29b-3p group (Fig. 6n). Total IκBα remained unchanged after the transfection of miR-29b-3p (*P*>0.05) (data not shown). The opposite results were obtained when miR-29b-3p was inhibited (Fig. 6e, i-k, o, p). It was revealed that the HuR modulated by miR-29b-3p may contribute to inhibiting p65 expression in nucleus, thus deactivating the NF-κB signaling pathway in AML cells.

### miR-29b-3p reduces the activity of STAT signaling pathway in AML cells by targeting HuR

**Fig. 7:**
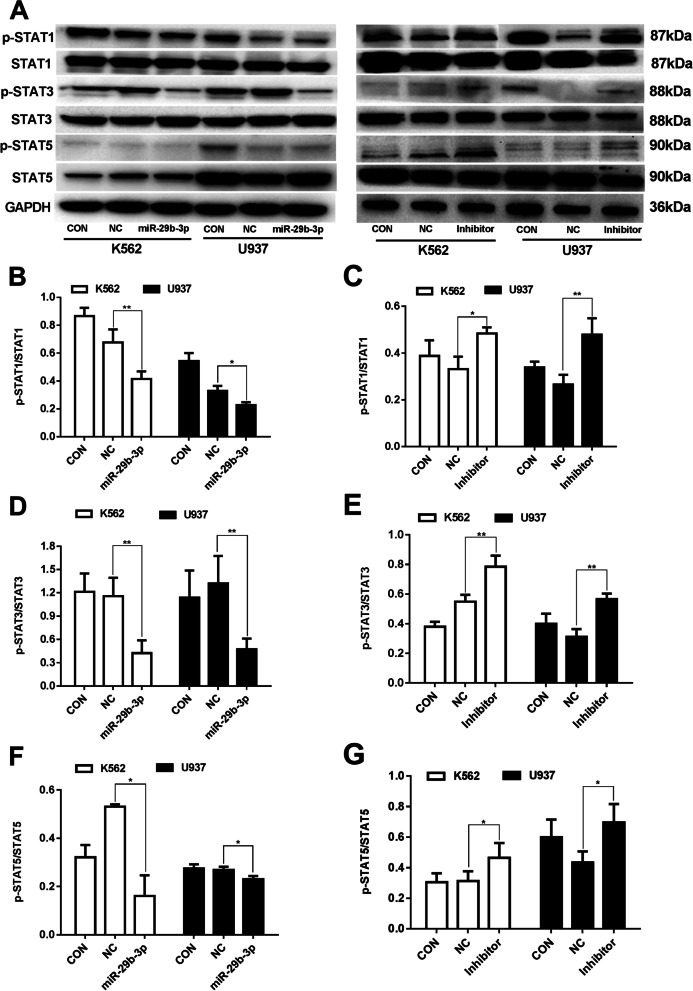
Effects of miR-29b-3p on the STAT signaling pathway protein by targeting HuR in AML cells. **a** STA1, STAT3, STAT5 and their phosphorylation proteins expression were detected by western blot. The original blots/gels are presented in Supplementary Figure 7. **b**-**g** Quantifications of phosphorylated STA1, STAT3, STAT5 were normalized to STA1, STAT3, STAT5 after miR-29b-3p over-expression and inhibition, respectively. (*P<0.05, **P<0.01, vs. NC group). The experiments were repeated at least three times

As shown in Fig. 7, up-regulated miR-29b-3p led to a sharp decline in the phosphorylation of STAT1 (*P*<0.05), STAT3 (*P*<0.01) and STAT5 (*P*<0.05) for K562 and U937 cells. However, total STAT1, STAT3 and STAT5 remained unchanged after the transfection of miR-29b-3p (*P*>0.05) (data not shown). The opposite results were obtained when miR-29b-3p was inhibited. According to the results, miR-29b-3p suppressed the phosphorylation of STAT1, STAT3 and STAT5, which is speculated to reduce the constitutive activation of STATs signaling in AML cells by targeting HuR.

## Discussion

AML is a hematopoietic stem cell disorder characterized by clonal expansion of abnormally differentiated myeloid lineage blasts. Though allogeneic hematopoietic cell transplantation (HCT) has been made great breakthroughs for AML patients’ therapy, molecular biomarkers in the management of AML still have the important role. Recently, post-transcriptional regulators of gene expression in malignant cancers have gained much attention. RNA-binding proteins (RBPs) play the important role in post-transcriptional expression of gene in hematopoietic. HuR, as a well-known molecule in RBPs, is involved in the post-transcriptional regulation of genes in almost many types of tumors, which is critical for neoplastic transformation and cancer development [2]. Although HuR is crucial in the post-transcriptional regulation of many genes, the regulation of its own function and expression remains obscure. Some studies have mentioned that miRNA may control the expression patterns of RBP in cancers [16], and previous evidences have confirmed that miR-29b mediated post-transcriptional regulation of HuR expression in cancer cells [14, 15]. Further research is warranted to better understand the role of miR-29b regulating by HuR in AML, so as to better identify potential therapeutic targets. The mature miR-29bs contains miR-29b-3p and miR-29b-5p, and the function of miR-29b is mainly played by miR-29b-3p [13]. In our current study, we first detected the expressions of HuR and miR-29b-3p in AML cells and analyzed the association between them. Our data showed that HuR was over-expression while miR-29b-3p was downregulation, and a significantly negative correlation between them in AML cells. We further focused on whether miR-29b-3p targeting HuR in AML cells. The prediction results of the bioinformatics database suggested that miR-29b-3p and HuR 3′UTR have multiple binding sites. We then verified that HuR 3′UTR was specifically associated with miR-29b-3p using the dual luciferase reporter assay. Moreover, in order to observe the regulation of miR-29b-3p on HuR, we performed RT-qPCR and Western blot after miR-29b-3p over- and down-expression, the results indicated that miR-29b-3p over-expression suppressed both HuR mRNA and protein expressions. Conversely, miR-29b-3p down-expression significantly increased HuR expressions. These findings demonstrated that HuR was a downstream target of miR-29b-3p in AML cells.

According to research reports, miR-29b is gaining prominence because of its emerging roles in a variety of physiological and pathological progresses including cell growth and differentiation, aging, transfer, immune regulation and cell cycle regulation and apoptosis [16–20]. The diagnostic and therapeutic implications of miR-29b has become a research hotspot in multiple malignancies especially hematological malignancies. In this study, miR-29b-3p was recognized as one of the critical factors. When we over- and down- regulate the expression of miR-29b-3p, we found that miR-29b-3p played a functional role in inhibiting the malignant biological activity including proliferation, invasion and migration of AML cells. Our results are consistent with the research report of the effect of miR-29b in AML carried out by Garzon R et al. in vivo and vitro [13].

Many studies have demonstrated that certain miRNAs differentially expressed in cancer cells are linked to the apoptotic death, disturbed cell cycle or the invasiveness and migration of these cells and to the progression of cancers through mechanisms associated with RBPs [21, 22]. Normally, HuR is located in the nuclei, however, stimuli (e.g., hypoxia, glucose deprivation, chemotherapy) in the tumor microenvironment will cause HuR relocation from the nucleus to the cytoplasm and promote HuR to bind to U- or AU-rich sequences in the 3′UTR of target mRNAs which confer cellular responses including cell growth, apoptosis and cell cycle [23]. There is an overlap in function between HuR and miR-29b-3p, hinting a potential close association between them. Whether miR-29b-3p regulates the malignant biological activity of AML cells was mediated by its downstream target HuR?To address this concern, we further silenced the expression of HuR artificially, and the results showed that down-regulating HuR in AML cells could inhibit the viability and invasion and migration ability of AML cells, and promote apoptosis. And the weakening of all malignant biological behaviors was restored by co-transfection with miR-29b-3p inhibitor. Therefore, HuR was the direct target of miR-29b-3p in AML cells not only structurally but also functionally.

It has been reported that the diverse functions of HuR in cancer development and progression are strongly associated with regulations of the stability or translation of target mRNAs that encode multiple cancer-related proteins [2, 8]. To assess the potential molecular pathway that how miR-29b-3p mediated HuR expression involves in the malignant transformation of AML cells, further experiments on localization, expression and activity of related pathway proteins after regulating the expression of miR-29b-3p were implemented in our study. One of the most central findings of the current study was that miR-29b-3p induced HuR inhibition suppressed oncogenic activation of the NF-κB and JAK/STAT signaling pathways.

NF-κB transcriptionally regulates relevant important genes controlling cell differentiation, proliferation, cell cycle, apoptosis and invasion to affect tumor progression. Previous studies have clearly proven that NF-κB signaling pathway often shows an abnormally high activation in AML cell lines [24]. Just as continuous activation of NF-κB may activate anti-apoptotic proteins Bcl-2 and Bcl-xL, suppressing NF-κB activity may activate its pre-apoptotic signals, thus increasing chemotherapy sensitivity of AML [25]. Classical NF-κB activation, whose main effector is RelA (p65)/p50, is usually a rapid and transient response to a wide range of stimuli. The conventional mechanism of triggering the NF-κB signaling pathway is associated with NF-κB and its inhibitor IκBα. Previous evidence has revealed that HuR can bind specifically to the IκBα mRNA 3'UTR to modulate the translation of IκBα mRNA leading to a change in NF-κB protein expressions [7], which in turn down-regulates NF-κB in the nucleus.

In addition, Mertens C et al. report that unphosphorylated STAT1 and STAT3 in the JAK/STAT signaling can be activated by the binding of NF-κB transcription factors [26]. The JAK/STAT pathway is also related to the formation of the blood system and immune responses, which can transmit anti-apoptotic, proliferation, differentiation [27]. Indeed, the NF-κB and JAK-STAT pathways possibly have exhibited constitutive signaling activities and hypersensitivity to cytokine stimulation. Sustained activation of NF-κB can initiate STAT3 phosphorylation on tyrosine residues dependent on the NF-κB-induced production of IL-6 which is a target gene of both NF-κB and STAT3 [28, 29]. The crosstalk between the JAK/STAT and NF-κB signal pathways may expose interlinked feedbacks for controlling cell fate decisions in cancer cellular populations. Consistent with this notion, the correlation between STAT5 and p65/RELA phosphorylation has also been observed in myelofibrosis and secondary AML [30]. Furthermore, miRNAs have been reported to prevent cancer progression through inactivating the JAK/STAT signaling pathway [31–33]. Unexpectedly, a previous study demonstrated that miR-29b reduced NF-κB activity which was functionally related to STAT3 [34]. In our study, HuR inhibition induced by miR-29b-3p over-expression resultantly decreased nuclear p65 activation and expression to make consistent suppression of the phosphorylation of the associated molecules in the NF-κB and JAK/STAT signal pathways. This indicates possible connections between miR-29b-3p mediated HuR inhibition and the crosstalk between the NF-κB and JAK/STAT pathways.

## Conclusions

In summary, Our study illustrates that miR-29b-3p affects the malignant biological activity of AML cells by targeting its downstream HuR, and involves the participation of NF-κB and JAK/STAT signaling pathways. However, the underlying correlations between this HuR inhibition and the crosstalk between the two signalings need to be verified in further intensive studies. Although miRNA-based therapeutic methods are still in their infancy, miR-29b-3p mediated HuR inhibition will inspire more promising therapeutic strategies for AML.

## Supplementary Information

**Additional file 1: Supplementary figure 1.** Original gels for all western blots in Figure 1D. Original gel image measuring immunopositivity against HuR in AML cells and healthy normal control. GAPDH was used as loading control. Bands used in the manuscript have been boxed in red. Red arrows represent protein markers.

**Additional file 2: Supplementary figure 2.** Original gels for all western blots in Figure 2H. Original gel image measuring immunopositivity against HuR in K562 and U937 cells after miR-29b-3p overexpression and was inhibited. GAPDH was used as loading control. Bands used in the manuscript have been boxed in red. Red arrows represent protein markers.

**Additional file 3: Supplementary figure 3.** Original gels for all western blots in Figure 3E. Original gel image measuring immunopositivity against Bcl-2 and Bax in K562 and U937 cells after miR-29b-3p overexpression. GAPDH was used as loading control. Bands used in the manuscript have been boxed in red. Red arrows represent protein markers.

**Additional file 4: Supplementary figure 4.** Original gels for all western blots in Figure 4E. Original gel image measuring immunopositivity against Bcl-2 and Bax in K562 and U937 cells after miR-29b-3p was inhibited. GAPDH was used as loading control. Bands used in the manuscript have been boxed in red. Red arrows represent protein markers.

**Additional file 5: Supplementary figure 5.** Original gels for all western blots in Figure 5B and 5F. Original gel image measuring immunopositivity against HuR, Bcl-2 and Bax in K562 and U937 cells after HuR down-regulation and rescued by miR-29b-3p inhibitor. GAPDH was used as loading control. Bands used in the manuscript have been boxed in red. Red arrows represent protein markers.

**Additional file 6: Supplementary figure 6.** Original gels for all western blots in Figure 6E and 6L. Original gel image measuring immunopositivity against Nucleus p65 , Cytoplasm p65, p-p65, p65, p-IκBα, IκBα in K562 and U937 cells after miR-29b-3p overexpression and was inhibited. PCNA was used as loading control for Nucleus p65. GAPDH was used as loading control for Cytoplasm p65, total P65, p-IκBα, total IκBα. Bands used in the manuscript have been boxed in red. Red arrows represent protein markers.

**Additional file 7: Supplementary figure 7.** Original gels for all western blots in Figure 7A. Original gel image measuring immunopositivity against p-STAT1, STAT1, p-STAT3, STAT3, p-STAT5 and STAT5 in K562 and U937 cells after miR-29b-3p overexpression and was inhibited. GAPDH was used as loading control. Bands used in the manuscript have been boxed in red. Red arrows represent protein markers.

**Additional file 8: Supplementary figure 8.** Statistical analysis of apoptosis-related protein expression levels and invasion cell numbers. **A-C.** The Bcl-2 and Bax protein levels and ratio of Bcl-2 to Bax after miR-29b-3p over-expression. **D-F.** The Bcl-2 and Bax protein levels and ratio of Bcl-2 to Bax after miR-29b-3p was inhibited. **G-I.** The Bcl-2 and Bax protein levels and ratio of Bcl-2 to Bax after transfection with HuR siRNA and co-transfection with miR-29b-3p inhibitor. **J.** The number of invasion cells after miR-29b-3p over-expression. **K.** The number of invasion cells after after miR-29b-3p was inhibited. **L.**The number of invasion cells after transfection with HuR siRNA and co-transfection with miR-29b-3p inhibitor.

**Additional file 9: Supplementary Table 1.** Cell cycle ratio of AML cells in each group after overexpression of miR-29b-3p. ** represents *P*<0.01 vs NC group.

**Additional file 10: Supplementary Table 2.** Cell cycle ratio of AML cells in each group after miR-29b-3p inhibition

**Additional file 11: Supplementary Table 3.** Cell cycle ratio of AML cells in each group after HuR down-regulation and recovery with miR-29b-3p inhibition. ** represents *P*<0.01 vs HuR-NC group. # # represents *P*<0.01 vs HuR-KD group.

**Additional file 12: Supplementary Table 4.** Apoptosis rates of AML cells in each group after overexpression of miR-29b-3p. ** represents *P*<0.01 vs NC group.

**Additional file 13**:** Supplementary Table 5.** Apoptosis rates of AML cells in each group after miR-29b-3p inhibition. ** represents *P*<0.01 vs NC group.

**Additional file 14: Supplementary Table 6.** Apoptosis rate of AML cells in each group after HuR down-regulation and recovery with miR-29b-3p inhibition. ** represents *P*<0.01 vs HuR-NC group. # # represents *P*<0.01 vs HuR-KD group.

## Data Availability

The authors declare that all data used or analysed during the current study are available on reasonable request.

## Abbreviations

HuR/ELAVL1: Embryonic lethal abnormal vision 1
AML: Acute myeloid leukemia
Bcl-2: B-cell lymphoma-2
Bax: Bcl-2-Associated X
PCNA: Proliferating Cell Nuclear Antigen
STAT: Signal transducers and activators of transcription

## References

[1] Zhou Y, Zhang X, Guan Y (2015). Human antigen R: A novel therapeutic target for diabetic nephropathy?. J Diabetes.

[2] Wang J, Guo Y, Chu H, Guan Y, Bi J, Wang B (2013). Multiple functions of the RNA-Binding protein HuR in cancer progression, treatment responses and prognosis. Int J Mol Sci.

[3] Wang J, Zhao W, Guo Y, Zhang B, Xie Q, Xiang D, Gao J, Wang B, Chen Z (2009). The expression of RNA-binding protein HuR in non-small cell lung cancer correlates with vascular endothelial growth factor-C expression and lymph node metastasis. Oncol.

[4] Lim SJ, Lee SH, Sun HJ, Song JY, Choi SI (2009). Cytoplasmic expression of HuR is related to cyclooxygenase-2 expression in colon cancer. Cancer Res Treat.

[5] Liang PI, Li WM, Wang YH, Wu TF, Li CF (2012). HuR cytoplasmic expression is associated with increased cyclin A expression and poor outcome with upper urinary tract urothelial carcinoma. BMC Cancer.

[6] López de Silanes I, Lal A, Gorospe M (2005). HuR: post-transcriptional paths to malignancy. RNA Biol.

[7] Liu Y, Yu W (2014). Heat shock-mediated regulation of IκB-α at the post-transcriptional level by HuR. Mol Med Rep.

[8] Baou M, Norton JD, Murphy JJ (2011). AU-rich RNA binding proteins in hematopoiesis and leukemogenesis. Blood.

[9] Ghaedi H, Bastami M, Zare-Abdollahi D, Alipoor B, Movafagh A, Mirfakhraie R, Omrani MD, Masotti A (2015). Bioinformatics prioritization of SNPs perturbing microRNA regulation of hematological malignancy-implicated genes. Genomics.

[10] Tania MC, David DL, Eline V, Gert O, Linda S (2017). Specific depletion of leukemic stem cells: can micrornas make the difference?. Cancers.

[11] Ciccone M, Calin GA (2015). MicroRNAs in myeloid hematological malignancies. Current Genomics.

[12] Vezzali F, Grassilli S, Lambertini E, Brugnoli F, Patergnani S, Nika E, Piva R, Pinton P, Capitani S, Bertagnolo V (2018). Vav1 is necessary for PU.1 mediated upmodulation of miR-29b in acute myeloid leukaemia-derived cells. J Cell Mol Med.

[13] Garzon R, Heaphy CE, Havelange V, Fabbri M, Volinia S, Tsao T, Zanesi N, Kornblau SM, Marcucci G, Calin GA (2009). MicroRNA 29b functions in acute myeloid leukemia. Blood.

[14] Li Y, Chen G, Wang JY, Zou T, Liu L, Xiao L, Chung HK, Rao JN, Wang JY (2016). Post-transcriptional regulation of Wnt co-receptor LRP6 and RNA-binding protein HuR by miR-29b in intestinal epithelial cells. Biochem J.

[15] Balkhi MY, Iwenofu OH, Bakkar N, Ladner KJ, Chandler DS, Houghton PJ, London CA, Kraybill W, Perrotti D, Croce CM (2013). miR-29 acts as a decoy in sarcomas to protect the tumor suppressor A20 mRNA from degradation by HuR. Sci Signal.

[16] Ciafrè Silvia Anna, Galardi Silvia (2013). microRNAs and RNA-binding proteins: a complex network of interactions and reciprocal regulations in cancer. RNA Biology.

[17] Poudyal D, Cui X, Le PM, Hofseth AB, Windust A, Nagarkatti M, Nagarkatti PS, Schetter AJ, Harris CC, Hofseth LJ (2013). A key role of microRNA-29b for the suppression of colon cancer cell migration by American ginseng. PLoS One.

[18] Chen L, Li Q, Wang J, Jin S, Zheng H, Lin J, He F, Zhang H, Ma S, Mei J (2017). MiR-29b-3p promotes chondrocyte apoptosis and facilitates the occurrence and development of osteoarthritis by targeting PGRN. J Cell Mol Med.

[19] Wang H, Ding Q, Wang M, Guo M, Zhao Q (2019). miR-29b inhibits the progression of multiple myeloma through downregulating FOXP1. Hematol.

[20] Zhao X, Liu Y, Li Z, Zheng S, Chen R (2018). Linc00511 acts as a competing endogenous RNA to regulate VEGFA expression through sponging hsa-miR-29b-3p in pancreatic ductal adenocarcinoma. J Cell Mol Med.

[21] Abdelmohsen K, Kim MM, Srikantan S, Mercken EM, Brennan SE, Wilson GM, De Cabo R, Gorospe M (2010). miR-519 suppresses tumor growth by reducing HuR levels. Cell Cycle.

[22] Abdelmohsen K, Srikantan S, Kuwano Y, Gorospe M (2008). miR-519 reduces cell proliferation by lowering RNA-binding protein HuR levels. Proc Natl Acad Sci U S A.

[23] Sueyoshi T, Kawasaki T, Kitai Y, Ori D, Akira S, Kawai T (2018). Hu antigen R regulates antiviral innate immune responses through the stabilization of mRNA for polo-like kinase 2. J Immunol.

[24] Bosman Matthieu Cornelis Johannes, Schuringa Jan Jacob, Vellenga Edo (2016). Constitutive NF-κB activation in AML: Causes and treatment strategies. Crit Rev Oncol Hematol.

[25] Griessinger E, Frelin C, Cuburu N, Imbert V, Dageville C, Hummelsberger M, Sirvent N, Dreano M, Peyron JF (2008). Preclinical targeting of NF-kappaB and FLT3 pathways in AML cells. Leuk.

[26] Mertens C, Darnell JE (2007). Snapshot: JAK-STAT signaling. Cell.

[27] Vainchenker W, Constantinescu SN (2013). JAK/STAT signaling in hematological malignancies. Oncog.

[28] Rozovski U, Harris DM, Li P, Liu Z, Jain P, Veletic I, Ferrajoli A, Burger J, Thompson P, Jain N, Wierda W, Keating MJ, Estrov Z (2017). Activation of the B-cell receptor successively activates NF-κB and STAT3 in chronic lymphocytic leukemia cells. Int J Cancer..

[29] Lee KS, Park JH, Lee S, Lim HJ, Choi HE, Park HY (2007). HB-EGF induces delayed STAT3 activation via NF-kappaB mediated IL-6 secretion in vascular smooth muscle cell. Biochim Biophys Acta.

[30] Fisher DAC, Malkova O, Engle EK, Miner CA, Fulbright MC, Behbehani GK, Collins TB, Bandyopadhyay S, Zhou A, Nolan GP, Oh ST (2017). Mass cytometry analysis reveals hyperactive NF Kappa B signaling in myelofibrosis and secondary acute myeloid leukemia. Leuk.

[31] Bi CL, Zhang YQ, Li B, Guo M, Fu YL (2019). MicroRNA-520a-3p suppresses epithelial-mesenchymal transition, invasion, and migration of papillary thyroid carcinoma cells via the JAK1-mediated JAK/STAT signaling pathway. J Cell Physiol..

[32] Zhang W, Wang Y, Zhu Z, Yan Z, Song B (2018). Propofol inhibits proliferation, migration and invasion of gastric cancer cells by up-regulating microRNA-195. Int J Biol Macromol.

[33] Teng Y, Ross JL, Cowell JK (2014). The involvement of JAK-STAT3 in cell motility, invasion, and metastasis. JAKSTAT.

[34] Lee H, Herrmann A, Deng JH, Kujawski M, Niu G, Li Z, Forman S, Jove R, Pardoll DM, Hua Y (2009). Persistently activated Stat3 maintains constitutive NF-kappaB activity in tumors. Cancer Cell.

