# Supplementary material for: miR-29b-3p suppresses the malignant biological behaviors of AML cells via inhibiting NF-κB and JAK/STAT signaling pathways by targeting HuR
Source: BMC Cancer. 2022 Aug 20;22:909. doi: 10.1186/s12885-022-09996-1 (PMC9392259; doi:10.1186/s12885-022-09996-1)
Supplement: Supplementary file 1 — Additional file 1: Supplementary figure 1. Original gels for all western blots in Figure 1D. Original gel image measuring immunopositivity against HuR in AML cells and healthy normal control. GAPDH was used as loading control. Bands used in the manuscript have been boxed in red. Red arrows represent protein markers. [file 12885_2022_9996_MOESM1_ESM.docx]

**Supplementary figure 1：Original gels for all western blots in Figure 1D**


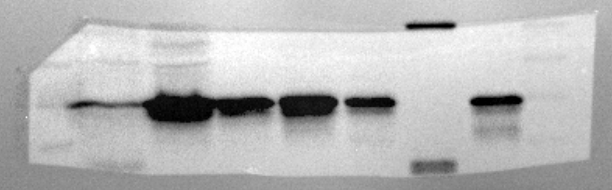


40KDa

35KDa

25KDa

HuR（36KDa）


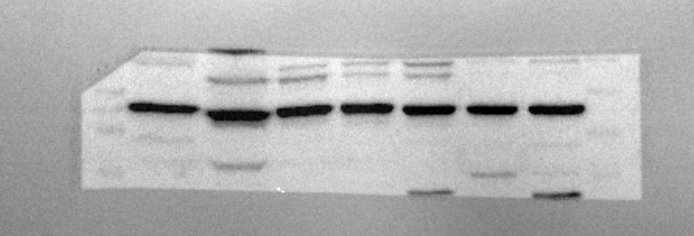


40KDa

35KDa

25KDa

GAPDH（36KDa）

U937

NB4

HL-60

K562/G01

K562

Kasumi-1

Normal

**Figure legend**: Original gel image measuring immunopositivity against HuR in AML cells and healthy normal control. GAPDH was used as loading control. Bands used in the manuscript have been boxed in red. Red arrows represent protein markers.
