# Supplementary material for: miR-29b-3p suppresses the malignant biological behaviors of AML cells via inhibiting NF-κB and JAK/STAT signaling pathways by targeting HuR
Source: BMC Cancer. 2022 Aug 20;22:909. doi: 10.1186/s12885-022-09996-1 (PMC9392259; doi:10.1186/s12885-022-09996-1)
Supplement: Supplementary file 3 — Additional file 3: Supplementary figure 3. Original gels for all western blots in Figure 3E. Original gel image measuring immunopositivity against Bcl-2 and Bax in K562 and U937 cells after miR-29b-3p overexpression. GAPDH was used as loading control. Bands used in the manuscript have been boxed in red. Red arrows represent protein markers. [file 12885_2022_9996_MOESM3_ESM.docx]

**Supplementary figure 3：Original gels for all western blots in Figure 3E**


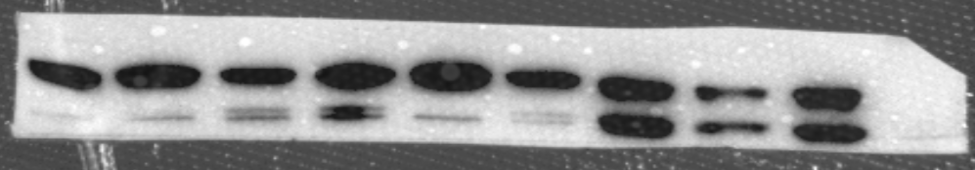


Bcl-2（26KDa）

35KDa

25KDa

25KDa

15KDa


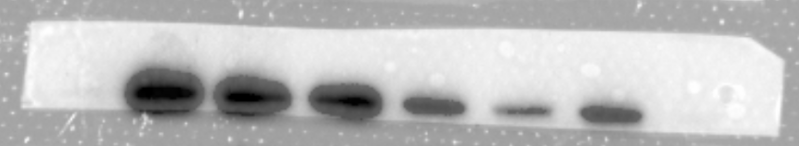


Bax（21KDa）


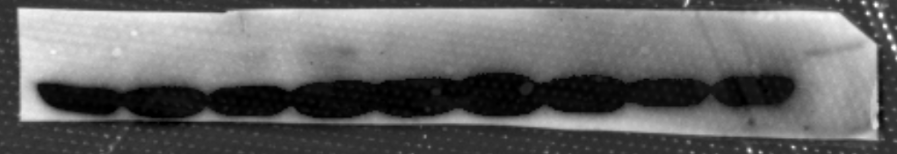


40KDa

35KDa

GAPDH（36KDa）

CON NC miR-29b-3p

CON NC miR-29b-3p

K562

U937

**Figure legend**: Original gel image measuring immunopositivity against Bcl-2 and Bax in K562 and U937 cells after miR-29b-3p overexpression. GAPDH was used as loading control. Bands used in the manuscript have been boxed in red. Red arrows represent protein markers.
