# Supplementary material for: miR-29b-3p suppresses the malignant biological behaviors of AML cells via inhibiting NF-κB and JAK/STAT signaling pathways by targeting HuR
Source: BMC Cancer. 2022 Aug 20;22:909. doi: 10.1186/s12885-022-09996-1 (PMC9392259; doi:10.1186/s12885-022-09996-1)
Supplement: Supplementary file 4 — Additional file 4: Supplementary figure 4. Original gels for all western blots in Figure 4E. Original gel image measuring immunopositivity against Bcl-2 and Bax in K562 and U937 cells after miR-29b-3p was inhibited. GAPDH was used as loading control. Bands used in the manuscript have been boxed in red. Red arrows represent protein markers. [file 12885_2022_9996_MOESM4_ESM.docx]

**Supplementary figure 4：Original gels for all western blots in Figure 4E**

Exposure time 20.3 seconds

Exposure time 2.2 seconds

25KDa

15KDa


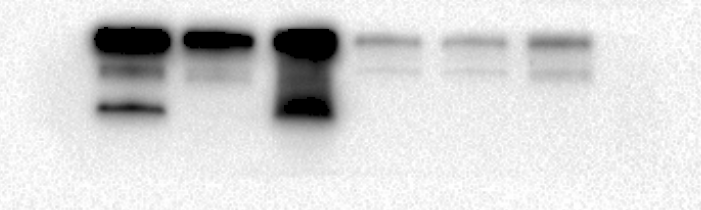

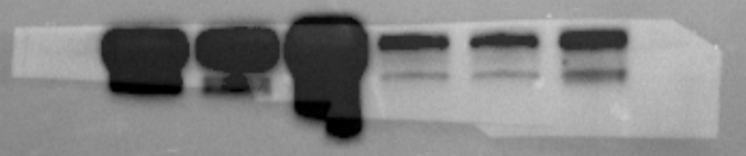


Bcl-2（26KDa）

CON NC Inhibitor

CON NC Inhibitor

K562

U937


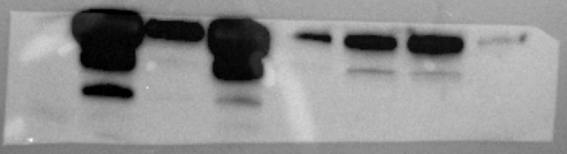


25KDa

15KDa

Bax（21KDa）

CON NC Inhibitor

K562


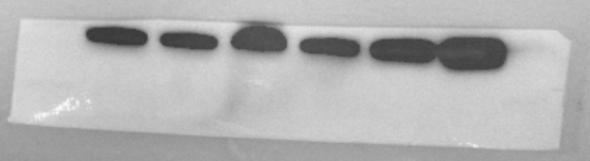


25KDa

15KDa

Bax（21KDa）

CON NC Inhibitor

U937


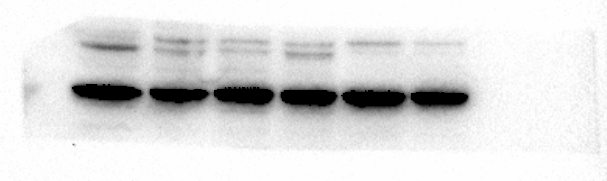


40KDa

35KDa

GAPDH（36KDa）

CON NC Inhibitor

CON NC Inhibitor

K562

U937

**Figure legend**: Original gel image measuring immunopositivity against Bcl-2 and Bax in K562 and U937 cells after miR-29b-3p was inhibited. GAPDH was used as loading control. Bands used in the manuscript have been boxed in red. Red arrows represent protein markers.
