# Supplementary material for: miR-29b-3p suppresses the malignant biological behaviors of AML cells via inhibiting NF-κB and JAK/STAT signaling pathways by targeting HuR
Source: BMC Cancer. 2022 Aug 20;22:909. doi: 10.1186/s12885-022-09996-1 (PMC9392259; doi:10.1186/s12885-022-09996-1)
Supplement: Supplementary file 5 — Additional file 5: Supplementary figure 5. Original gels for all western blots in Figure 5B and 5F. Original gel image measuring immunopositivity against HuR, Bcl-2 and Bax in K562 and U937 cells after HuR down-regulation and rescued by miR-29b-3p inhibitor. GAPDH was used as loading control. Bands used in the manuscript have been boxed in red. Red arrows represent protein markers. [file 12885_2022_9996_MOESM5_ESM.docx]

**Supplementary figure 5：Original gels for all western blots in Figure 5B and 5F**

**5B**


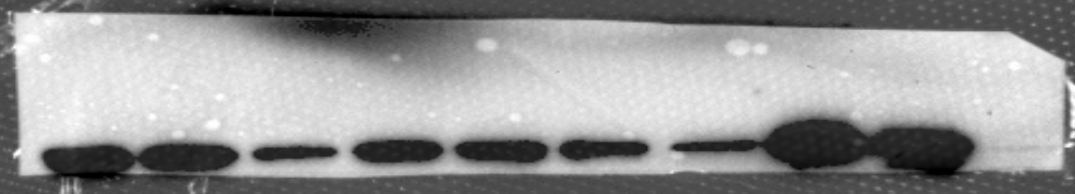


HuR（36KDa）


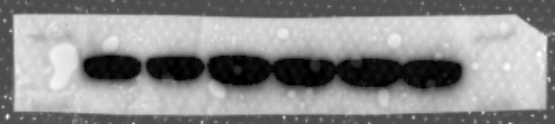


40KDa

35KDa

GAPDH（36KDa）

HuR-KD+

miR-29b-3p Inhibitor

CON NC

HuR-KD+

miR-29b-3p Inhibitor

CON NC

U937

K562

**5F**
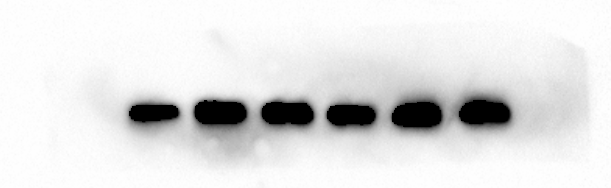


25KDa

15KDa

Bcl-2（26KDa）


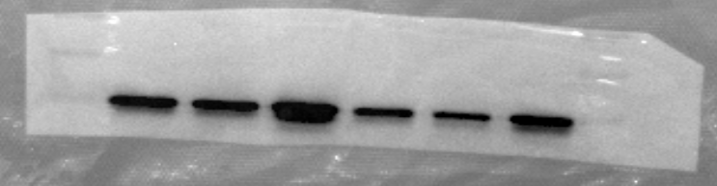


25KDa

15KDa

Bax（21KDa）


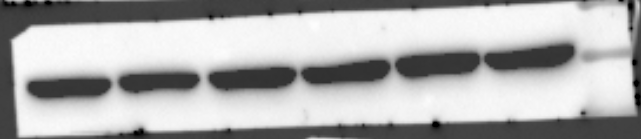
-

40KDa

35KDa

GAPDH（36KDa）

HuR-KD+

miR-29b-3p Inhibitor

CON NC

HuR-KD+

miR-29b-3p Inhibitor

CON NC

U937

K562

**Figure legend**: Original gel image measuring immunopositivity against HuR, Bcl-2 and Bax in K562 and U937 cells after HuR down-regulation and rescued by miR-29b-3p inhibitor. GAPDH was used as loading control. Bands used in the manuscript have been boxed in red. Red arrows represent protein markers.
