# Supplementary material for: miR-29b-3p suppresses the malignant biological behaviors of AML cells via inhibiting NF-κB and JAK/STAT signaling pathways by targeting HuR
Source: BMC Cancer. 2022 Aug 20;22:909. doi: 10.1186/s12885-022-09996-1 (PMC9392259; doi:10.1186/s12885-022-09996-1)
Supplement: Supplementary file 6 — Additional file 6: Supplementary figure 6. Original gels for all western blots in Figure 6E and 6L. Original gel image measuring immunopositivity against Nucleus p65 , Cytoplasm p65, p-p65, p65, p-IκBα, IκBα in K562 and U937 cells after miR-29b-3p overexpression and was inhibited. PCNA was used as loading control for Nucleus p65. GAPDH was used as loading control for Cytoplasm p65, total P65, p-IκBα, total IκBα. Bands used in the manuscript have been boxed in red. Red arrows represent protein markers. [file 12885_2022_9996_MOESM6_ESM.docx]

**Supplementary figure 6：Original gels for all western blots in Figure 6E and 6L**

**6E**


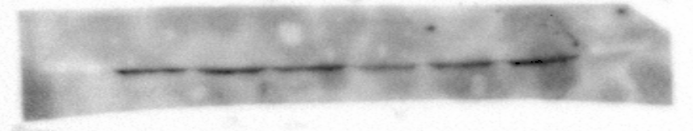


70KDa

Nucleus p65（65KDa）


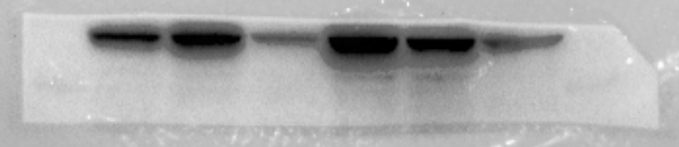


70KDa

55KDa

Cytoplasm p65（65KDa）

70KDa

55KDa


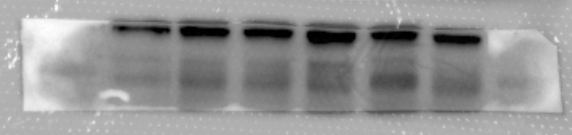


p65

（65KDa）


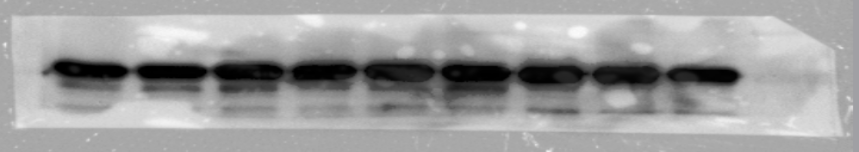


40KDa

35KDa

PCNA

（36KDa）


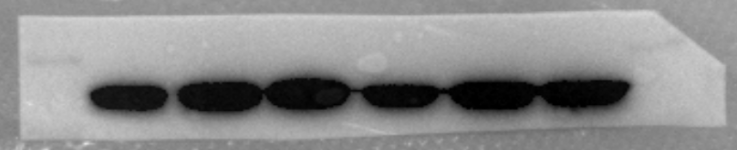


40KDa

35KDa

GAPDH（36KDa）

CON NC miR-29b-3p

CON NC miR-29b-3p

U937

K562


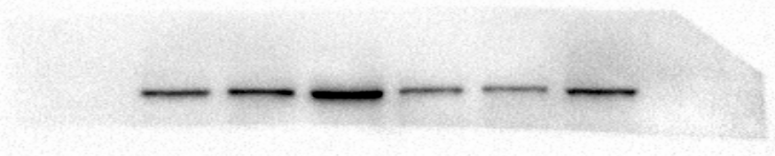


70KDa

55KDa

Nucleus p65（65KDa）


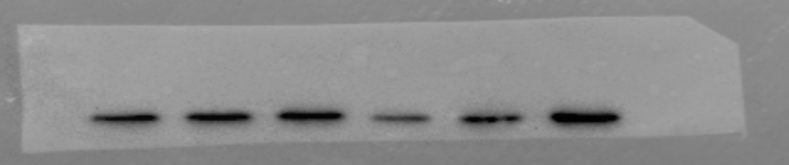


70KDa

55KDa

Cytoplasm p65（65KDa）


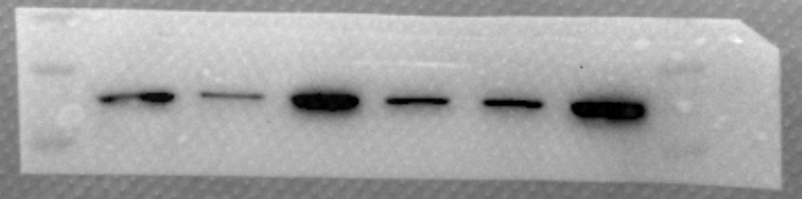


70KDa

55KDa

p65

（65KDa）


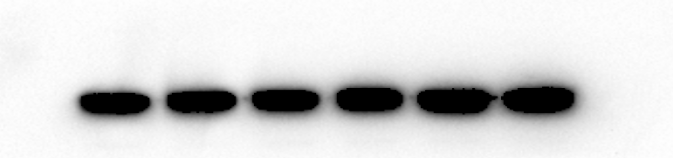


40KDa

35KDa

PCNA

（36KDa）

40KDa

35KDa


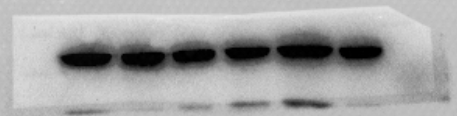


GAPDH（36KDa）

CON NC Inhibitor

CON NC Inhibitor

U937

K562

**6L**


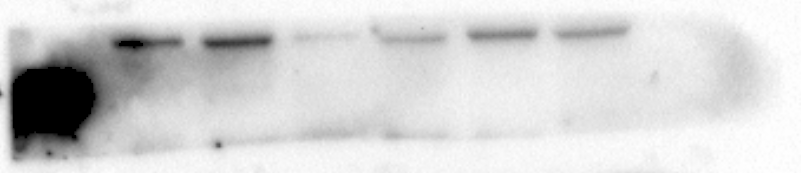


70KDa

55KDa

p-p65

（65KDa）


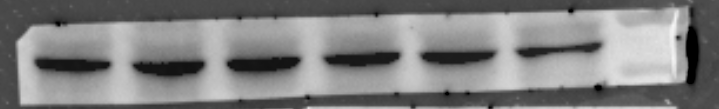


70KDa

55KDa

p65

（65KDa）


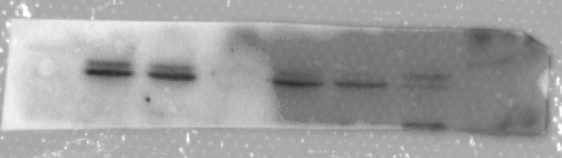


40KDa

35KDa

p-IκBα

（36KDa）


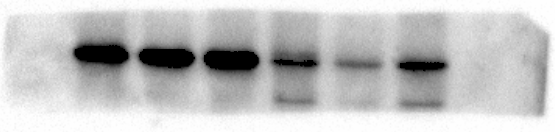


40KDa

35KDa

IκBα

（35KDa）


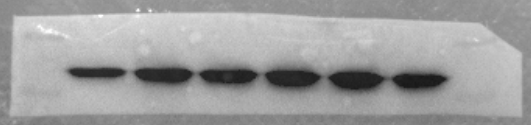


40KDa

35KDa

GAPDH（36KDa）

CON NC miR-29b-3p

CON NC miR-29b-3p

U937

K562


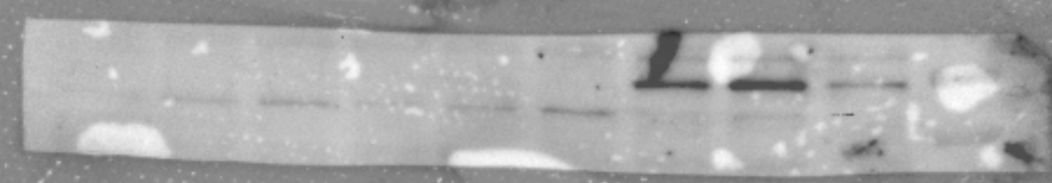


70KDa

55KDa

p-p65

（65KDa）


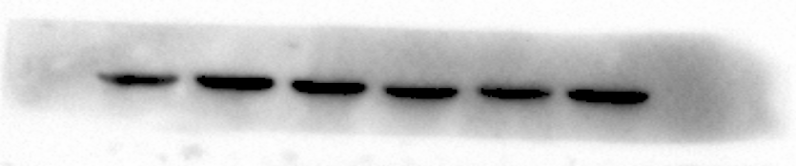


p65

（65KDa）

70KDa

55KDa


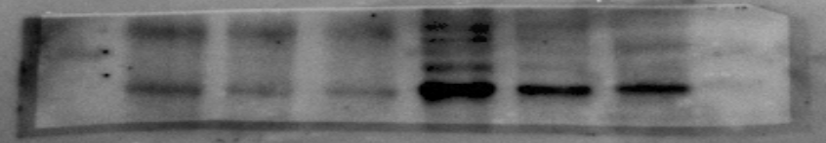


40KDa

35KDa

p-IκBα

（36KDa）


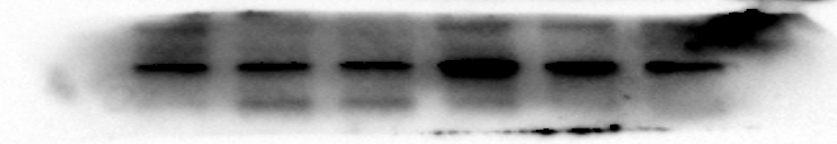


40KDa

35KDa

IκBα

（35KDa）


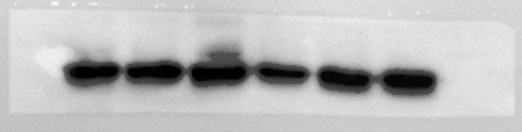


40KDa

35KDa

GAPDH（36KDa）

CON NC Inhibitor

CON NC Inhibitor

U937

K562

**Figure legend**: Original gel image measuring immunopositivity against Nucleus p65 , Cytoplasm p65, p-p65, p65, p-IκBα, IκBα in K562 and U937 cells after miR-29b-3p overexpression and was inhibited. PCNA was used as loading control for Nucleus p65. GAPDH was used as loading control for Cytoplasm p65, total P65, p-IκBα, total IκBα. Bands used in the manuscript have been boxed in red. Red arrows represent protein markers.
