# Supplementary material for: miR-29b-3p suppresses the malignant biological behaviors of AML cells via inhibiting NF-κB and JAK/STAT signaling pathways by targeting HuR
Source: BMC Cancer. 2022 Aug 20;22:909. doi: 10.1186/s12885-022-09996-1 (PMC9392259; doi:10.1186/s12885-022-09996-1)
Supplement: Supplementary file 7 — Additional file 7: Supplementary figure 7. Original gels for all western blots in Figure 7A. Original gel image measuring immunopositivity against p-STAT1, STAT1, p-STAT3, STAT3, p-STAT5 and STAT5 in K562 and U937 cells after miR-29b-3p overexpression and was inhibited. GAPDH was used as loading control. Bands used in the manuscript have been boxed in red. Red arrows represent protein markers. [file 12885_2022_9996_MOESM7_ESM.docx]

**Supplementary figure 7：Original gels for all western blots in Figure 7A**


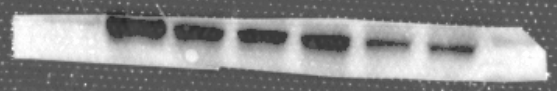


100KDa

p-STAT1

（87KDa）

70KDa


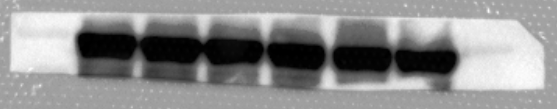


100KDa

STAT1

（87KDa）

70KDa


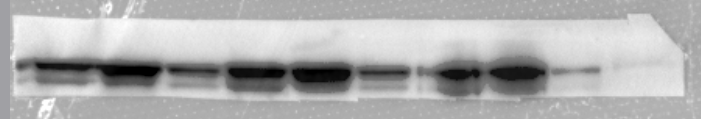


100KDa

p-STAT3

（88KDa）


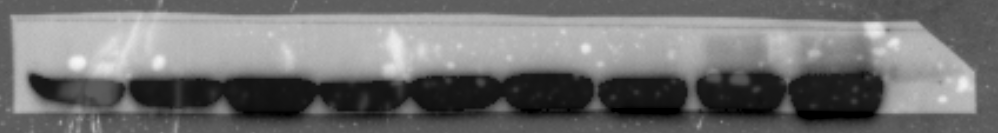


100KDa

STAT3

（88KDa）


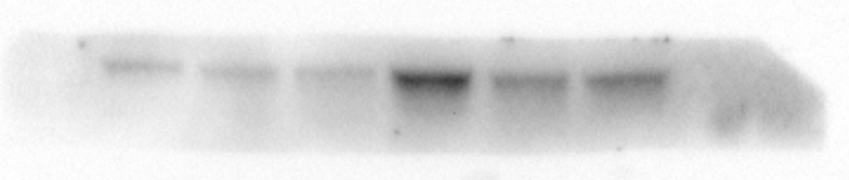


100KDa70KDa

p-STAT5

（90KDa）


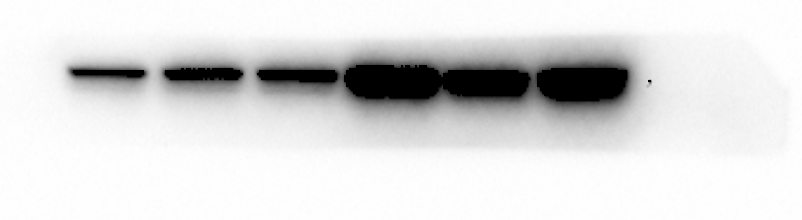


100KDa70KDa

STAT5

（90KDa）


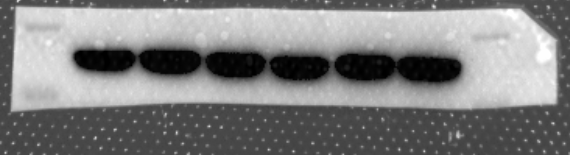


40KDa

35KDa

GAPDH（36KDa）

CON NC miR-29b-3p

CON NC miR-29b-3p

U937

K562


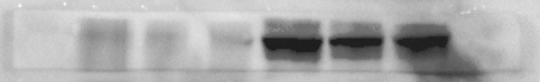


100KDa70KDa

p-STAT1

（87KDa）

CON NC Inhibitor

K562


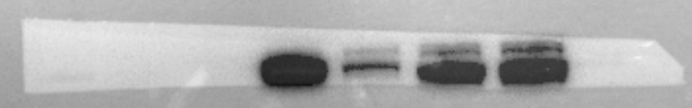


p-STAT1

（87KDa）

100KDa

U937

CON NC Inhibitor

100KDa70KDa


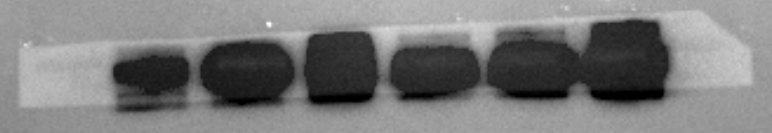


STAT1

（87KDa）


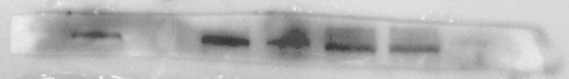

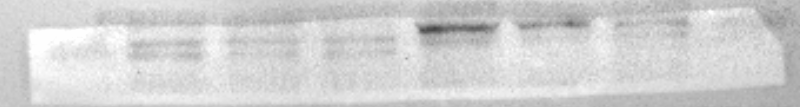

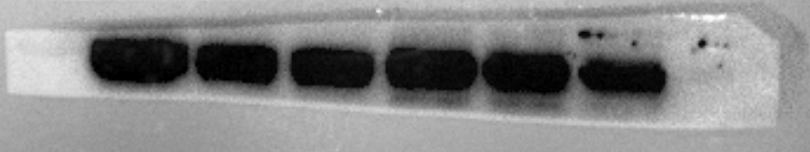

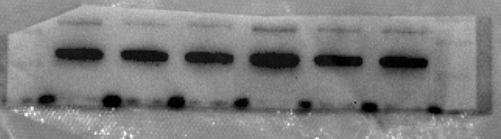

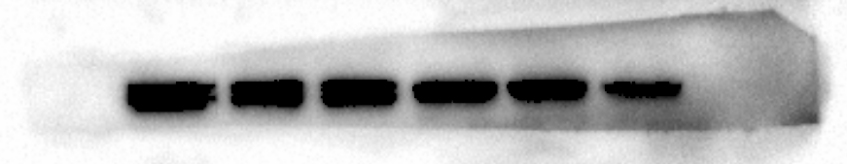


100KDa70KDa

100KDa70KDa

100KDa

100KDa70KDa

40KDa

35KDa

p-STAT5

（90KDa）

STAT5

（90KDa）

p-STAT3

（88KDa）

STAT3

（88KDa）

GAPDH（36KDa）

U937

K562

CON NC Inhibitor

CON NC Inhibitor

**Figure legend**: Original gel image measuring immunopositivity against p-STAT1, STAT1, p-STAT3, STAT3, p-STAT5 and STAT5 in K562 and U937 cells after miR-29b-3p overexpression and was inhibited. GAPDH was used as loading control.Bands used in the manuscript have been boxed in red. Red arrows represent protein markers.
