# Supplementary material for: miR-29b-3p suppresses the malignant biological behaviors of AML cells via inhibiting NF-κB and JAK/STAT signaling pathways by targeting HuR
Source: BMC Cancer. 2022 Aug 20;22:909. doi: 10.1186/s12885-022-09996-1 (PMC9392259; doi:10.1186/s12885-022-09996-1)
Supplement: Supplementary file 8 — Additional file 8: Supplementary figure 8. Statistical analysis of apoptosis-related protein expression levels and invasion cell numbers. A-C. The Bcl-2 and Bax protein levels and ratio of Bcl-2 to Bax after miR-29b-3p over-expression. D-F. The Bcl-2 and Bax protein levels and ratio of Bcl-2 to Bax after miR-29b-3p was inhibited. G-I. The Bcl-2 and Bax protein levels and ratio of Bcl-2 to Bax after transfection with HuR siRNA and co-transfection with miR-29b-3p inhibitor. J. The number of invasion cells after miR-29b-3p over-expression. K. The number of invasion cells after after miR-29b-3p was inhibited. L.The number of invasion cells after transfection with HuR siRNA and co-transfection with miR-29b-3p inhibitor. [file 12885_2022_9996_MOESM8_ESM.docx]

**Supplementary Figure 8: Statistical analysis of apoptosis-related protein expression levels and invasion cell numbers.**


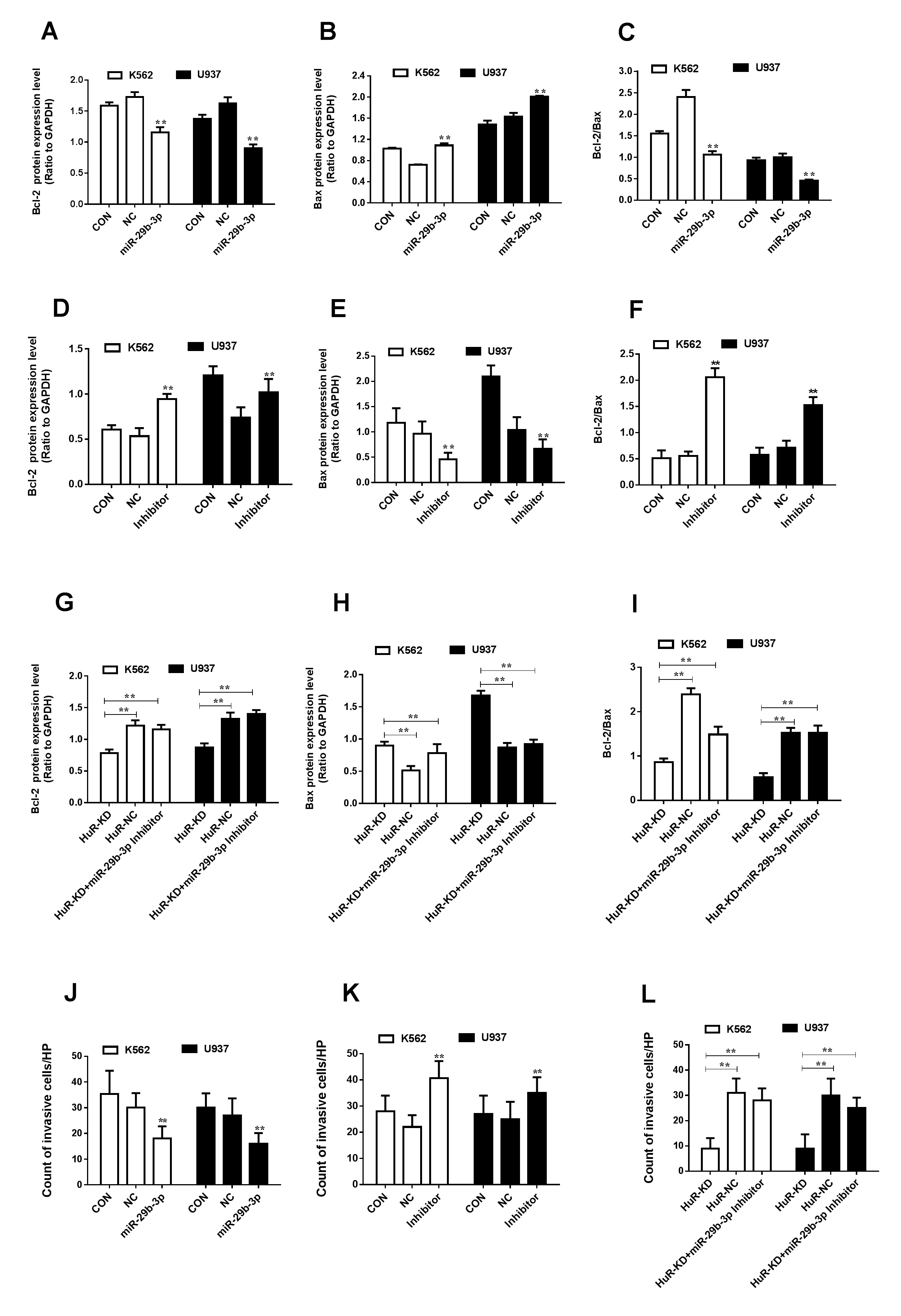


(A-C) The Bcl-2 and Bax protein levels and ratio of Bcl-2 to Bax after miR-29b-3p over-expression. (D-F) The Bcl-2 and Bax protein levels and ratio of Bcl-2 to Bax after miR-29b-3p was inhibited. (G-I) The Bcl-2 and Bax protein levels and ratio of Bcl-2 to Bax after transfection with HuR siRNA and co-transfection with miR-29b-3p inhibitor. (J) The number of invasion cells after miR-29b-3p over-expression. (K) The number of invasion cells after after miR-29b-3p was inhibited. (L)The number of invasion cells after transfection with HuR siRNA and co-transfection with miR-29b-3p inhibitor.
