# Supplementary material for: miR-29b-3p suppresses the malignant biological behaviors of AML cells via inhibiting NF-κB and JAK/STAT signaling pathways by targeting HuR
Source: BMC Cancer. 2022 Aug 20;22:909. doi: 10.1186/s12885-022-09996-1 (PMC9392259; doi:10.1186/s12885-022-09996-1)
Supplement: Supplementary file 9 — Additional file 9: Supplementary Table 1. Cell cycle ratio of AML cells in each group after overexpression of miR-29b-3p. ** represents P<0.01 vs NC group. [file 12885_2022_9996_MOESM9_ESM.docx]

**Supplementary Table 1：Cell cycle ratio of AML cells in each group after overexpression of miR-29b-3p**

| Group | Phase G1/G0（%） | Phase S（%） | Phase G2/M（%） |
| --- | --- | --- | --- |
| K562-CON | 16.933±1.651 | 79.520±1.306 | 3.547±0.371 |
| K562-NC | 31.397±0.617 | 66.540±0.322 | 2.063±0.542 |
| K562-miR-29b-3p | 35.347±0.709**^**^ (***P*=0.000**)** | 59.320±1.182**^**^**  **(***P*=0.000**)** | 5.333±0.544**^**^**  **(***P*=0.001**)** |
| U937-CON | 38.257±1.236 | 56.037±1.565 | 5.707±0.674 |
| U937-NC | 38.400±0.420 | 55.933±0.888 | 5.667±0.571 |
| U937-miR-29b-3p | 42.724±0.259**^**^**  **(***P*=0.000**)** | 50.873±0.413**^**^**  **(***P*=0.000**)** | 6.403±0.356  **(***P*=0.529**)** |

** represents *P*<0.01 vs NC group.
