# Supplementary material for: miR-29b-3p suppresses the malignant biological behaviors of AML cells via inhibiting NF-κB and JAK/STAT signaling pathways by targeting HuR
Source: BMC Cancer. 2022 Aug 20;22:909. doi: 10.1186/s12885-022-09996-1 (PMC9392259; doi:10.1186/s12885-022-09996-1)
Supplement: Supplementary file 10 — Additional file 10: Supplementary Table 2. Cell cycle ratio of AML cells in each group after miR-29b-3p inhibition [file 12885_2022_9996_MOESM10_ESM.docx]

**Supplementary Table 2：Cell cycle ratio of AML cells in each group after miR-29b-3p inhibition**

| Group | Phase G1/G0（%） | Phase S（%） | Phase G2/M（%） |
| --- | --- | --- | --- |
| K562-CON | 33.450±4.136 | 56.900±2.655 | 9.650±1.557 |
| K562-NC | 37.663±1.422 | 58.724±0.861 | 3.613±0.999 |
| K562-inhibitor | 36.263±1.829  **(***P*=0.688**)** | 59.607±2.082  **(***P*=0.860**)** | 4.130±0.417  **(***P*=0.949**)** |
| U937-CON | 55.040±1.741 | 41.493±2.241 | 3.467±0.773 |
| U937-NC | 54.980±5.445 | 42.844±4.678 | 2.176±1.024 |
| U937-inhibitor | 54.850±1.882  **(***P*=0.998**)** | 40.513±1.820  **(***P*=0.565**)** | 4.637±0.536  **(***P*=0.530**)** |
