# Supplementary material for: miR-29b-3p suppresses the malignant biological behaviors of AML cells via inhibiting NF-κB and JAK/STAT signaling pathways by targeting HuR
Source: BMC Cancer. 2022 Aug 20;22:909. doi: 10.1186/s12885-022-09996-1 (PMC9392259; doi:10.1186/s12885-022-09996-1)
Supplement: Supplementary file 11 — Additional file 11: Supplementary Table 3. Cell cycle ratio of AML cells in each group after HuR down-regulation and recovery with miR-29b-3p inhibition. ** represents P<0.01 vs HuR-NC group. # # represents P<0.01 vs HuR-KD group. [file 12885_2022_9996_MOESM11_ESM.docx]

**Supplementary Table 3：Cell cycle ratio of AML cells in each group after HuR down-regulation and recovery with miR-29b-3p inhibition**

| Group | Phase G1/G0（%） | Phase S（%） | Phase G2/M（%） |
| --- | --- | --- | --- |
| K562-HuR-KD | 40.484±0.843**^**^**  **(***P*=0.000**)** | 51.161±1.001**^**^**  **(***P*=0.000**)** | 8.354±1.479**^**^**  **(***P*=0.001**)** |
| K562-HuR-NC | 31.397±0.617 | 64.225±0.987 | 4.378±0.625 |
| K562-HuR-KD+miR-29b-3p inhibitor | 34.647±0.965 ^# #^  **(***P*=0.000**)** | 61.372±0.839 ^# #^  **(***P*=0.000**)** | 3.981±1.542 ^# #^  **(***P*=0.000**)** |
| U937-HuR-KD | 45.399±0.548**^**^**  **(***P*=0.000**)** | 46.586±1.526**^**^**  **(***P*=0.000**)** | 8.015±1.377  **(***P*=0.135**)** |
| U937-HuR-NC | 38.403±1.269 | 55.573±1.519 | 6.024±1.885 |
| U937-HuR-KD+miR-29b-3p inhibitor | 40.539±0.623 ^# #^  **(***P*=0.000**)** | 53.575±0.572 ^# #^  **(***P*=0.000**)** | 5.885±0.567  **(***P*=0.104**)** |

** represents *P*<0.01 vs HuR-NC group. # # represents *P*<0.01 vs HuR-KD group.
