# Supplementary material for: miR-29b-3p suppresses the malignant biological behaviors of AML cells via inhibiting NF-κB and JAK/STAT signaling pathways by targeting HuR
Source: BMC Cancer. 2022 Aug 20;22:909. doi: 10.1186/s12885-022-09996-1 (PMC9392259; doi:10.1186/s12885-022-09996-1)
Supplement: Supplementary file 12 — Additional file 12: Supplementary Table 4. Apoptosis rates of AML cells in each group after overexpression of miR-29b-3p. ** represents P<0.01 vs NC group. [file 12885_2022_9996_MOESM12_ESM.docx]

**Supplementary Table 4：Apoptosis rates of AML cells in each group after overexpression of miR-29b-3p**

| Group | Early apoptosis rate % | Late apoptosis rate % | Total apoptosis rate % |
| --- | --- | --- | --- |
| K562-CON | 6.433±0.115 | 0.467±0.058 | 6.900±0.173 |
| K562-NC | 6.633±0.252 | 0.300±0.000 | 6.933±0.252 |
| K562-miR-29b-3p | 14.033±0.578**^**^**  **(***P*=0.000**)** | 0.267±0.058  (*P*=0.955) | 14.300±0.575**^**^**  (*P*=0.000) |
| U937-CON | 4.733±0.208 | 1.967±0.577 | 6.700±0.265 |
| U937-NC | 4.400±0.173 | 2.400±0.000 | 6.800±0.173 |
| U937-miR-29b-3p | 5.967±0.208**^**^**  **(***P*=0.000**)** | 3.000±0.100**^**^**  **(***P*=0.003**)** | 8.967±0.289**^**^**  **(***P*=0.000**)** |

** represents *P*<0.01 vs NC group.
