# Supplementary material for: miR-29b-3p suppresses the malignant biological behaviors of AML cells via inhibiting NF-κB and JAK/STAT signaling pathways by targeting HuR
Source: BMC Cancer. 2022 Aug 20;22:909. doi: 10.1186/s12885-022-09996-1 (PMC9392259; doi:10.1186/s12885-022-09996-1)
Supplement: Supplementary file 13 — Additional file 13: Supplementary Table 5. Apoptosis rates of AML cells in each group after miR-29b-3p inhibition. ** represents P<0.01 vs NC group. [file 12885_2022_9996_MOESM13_ESM.docx]

**Supplementary Table 5 ：Apoptosis rates of AML cells in each group after miR-29b-3p inhibition**

| Group | Early apoptosis rate % | Late apoptosis rate % | Total apoptosis rate % |
| --- | --- | --- | --- |
| K562-CON | 2.433±0.058 | 0.600±0.100 | 3.033±0.153 |
| K562-NC | 6.667±0.208 | 1.633±0.153 | 8.300±0.361 |
| K562-inhibitor | 3.233±0.058**^**^**  **(***P*=0.000**)** | 1.333±0.058  **(***P*=0.092**)** | 4.567±0.058**^**^**  **(***P*=0.000**)** |
| U937-CON | 2.100±0.265 | 0.667±0.153 | 2.767±0.306 |
| U937-NC | 9.467±0.153 | 1.933±0.404 | 11.400±0.529 |
| U937-inhibitor | 6.033±0.802**^**^**  **(***P*=0.000**)** | 1.700±0.265  **(***P*=0.833**)** | 7.733±0.907**^**^**  **(***P*=0.000**)** |

** represents *P*<0.01 vs NC group.
