# Supplementary material for: miR-29b-3p suppresses the malignant biological behaviors of AML cells via inhibiting NF-κB and JAK/STAT signaling pathways by targeting HuR
Source: BMC Cancer. 2022 Aug 20;22:909. doi: 10.1186/s12885-022-09996-1 (PMC9392259; doi:10.1186/s12885-022-09996-1)
Supplement: Supplementary file 14 — Additional file 14: Supplementary Table 6. Apoptosis rate of AML cells in each group after HuR down-regulation and recovery with miR-29b-3p inhibition. ** represents P<0.01 vs HuR-NC group. # # represents P<0.01 vs HuR-KD group. [file 12885_2022_9996_MOESM14_ESM.docx]

**Supplementary Table 6：Apoptosis rate of AML cells in each group after HuR down-regulation and recovery with miR-29b-3p inhibition**

| Group | Early apoptosis rate% | Late apoptosis rate % | Total apoptosis rate % |
| --- | --- | --- | --- |
| K562-HuR-KD | 9.508±0.162^**^  **(***P*=0.000**)** | 6.154±0.158^**^  **(***P*=0.000**)** | 15.662±0.287^**^  **(***P*=0.000**)** |
| K562-HuR-NC | 6.599±0.221 | 0.681±0.066 | 7.280±0.234 |
| K562-HuR-KD+miR-29b-3p inhibitor | 6.295±0.157^# #^  **(***P*=0.000**)** | 0.859±0.087^# #^  **(***P*=0.000**)** | 7.154±0.210 ^# #^  **(***P*=0.000**)** |
| U937-HuR-KD | 11.679±0.167^**^  **(***P*=0.000**)** | 6.330±0.207^**^  **(***P*=0.000**)** | 18.008±0.372^**^  **(***P*=0.000**)** |
| U937-HuR-NC | 5.278±0.073 | 1.243±0.119 | 6.521±0.136 |
| U937-HuR-KD+miR-29b-3p inhibitor | 6.361±0.198 ^# #^  **(***P*=0.000**)** | 0.591±0.062^# #^  **(***P*=0.000**)** | 6.953±0.153 ^# #^  **(***P*=0.000**)** |

** represents *P*<0.01 vs HuR-NC group. # # represents *P*<0.01 vs HuR-KD group.
